# Supplementary material for: Development of a New Methodology for Dearomative Borylation of Coumarins and Chromenes and Its Applications to Synthesize Boron-Containing Retinoids
Source: Molecules. 2023 Jan 20;28(3):1052. doi: 10.3390/molecules28031052 (PMC9921500; doi:10.3390/molecules28031052)
Supplement: Supplementary file 1 [file molecules-28-01052-s001.zip › molecules-2150123-supplementary.pdf]

Supporting Information

Development of a new methodology for dearomative borylation of coumarins and chromenes and its applications to synthesize boron-containing retinoids

Bhaskar C. Das [1, 2,3,*], Pratik Yadav [1], Sasmita Das [1], Mariko Saito [3] and Todd Evans [2,*]

[1] Arnold and Marie Schwartz College of Pharmacy and Health Sciences, Long Island University, Brooklyn, NY-11201, USA.

[2] Department of Medicine and Pharmacological Sciences, Icahn School of Medicine at Mount Sinai, New York, NY 10029, USA.

[3] Department of Surgery, Weill Cornell Medical College of Cornell University, New York, NY 10065, USA.

[4] Nathan S. Kline Institute for Psychiatric Research, Orangeburg, NY 10962, USA.

Table of content

1. Experimental Selection 2

2. Synthesis of starting materials **6a-c** 2-3

3. Synthesis of compounds **2a-i, 4a-c, 5a-b and 7a-c** 3-7

4. Spectra of compounds 8-59

Experimental Section

General remarks:

Unless otherwise noted, all the catalysts and reagents were purchased from commercial suppliers, MilliporeSigma, USA and Fisher Scientific, USA and used as received. All the dry solvents were obtained from commercial sources and degassed according to standard procedure. Thin-layer chromatography (TLC) and column chromatography (CC) were performed with Kieselgel 60 F_{254} (Merck) and silica gel (Kieselgel 60, 230–400 mesh, Merck), respectively. Since all the compounds prepared contain aromatic ring, they were visualized and detected on TLC plates with UV light (short-wave, long-wave or both). NMR spectra were recorded on a BRUKER AVANCE NEO NANOBAY-USA (400 MHz) and $CDCl_3$, DMSO-d_6 and acetone-d_6 were used as a solvent.
Signal patterns are indicated as s, singlet; d, doublet; dd, doublet of doublets; t, triplet; m, multiplet; bs, broad singlet and bm, broad multiplet. Coupling constants (*J*) are given in hertz (Hz). Room temperature ranged from 25-30 °C during the reactions.

Synthesis of starting material 6a-c:

6,8-dichloro-2-phenyl-2H-chromene-3-carbonitrile 6a:

The compound was prepared by adding triflic acid (3.0 mmol) to a well-stirred solution of aldehyde **5d** (1.0 mmol), sodium azide (1.5 mmol) in acetonitrile (10 mL). The resulting mixture was stirred at room temperature and the progress of reaction was monitored by TLC. Upon completion, the solvent was removed under reduced pressure and the residue was extracted with ethyl acetate (3 X 15 mL). Ethyl acetate layer was washed with water, dried over anhydrous Na_2SO_4, concentrated and purified by column chromatography using hexane/ethyl acetate solvent system.

^{1}H NMR (400 MHz, $CDCl_3$): δ 6.05 (s, 1H), 7.05 (s, 1H), 7.25 (d, J = 10 Hz, 1H), 7.32 (s, 1H), 7.39-7.45 (m, 5H); ^{13}C NMR (100 MHz, $CDCl_3$): δ 109.2, 116.0, 121.2, 123.1, 126.1, 126.9, 129.1, 129.8, 132.6, 136.3, 147.7.

3-(3-(2-(trifluoromethoxy)phenyl)-1,2,4-oxadiazol-5-yl)-2H-chromen-2-one 6b:

In a 25 mL round bottom flask coumarin-3-carboxylic acid (1.0 mmol) and CDI (1.0 mmol) in DMF (5 mL) were heated at 70 °C for 30 minutes. To the resulting solution N'-hydroxy-2-(trifluoromethoxy)benzimidamide (1.2 mmol) was added. The reaction was stirred at 80 °C temperature and the progress of reaction was monitored by TLC. Upon completion, the reaction mixture was poured in ice-cold water (50 mL). The crude solid obtained was purified by column chromatography to obtain the desired product.

^{1}H NMR (400 MHz, DMSO-D_6): δ 7.47-7.55 (m, 2H), 7.66-7.71 (m, 2H), 7.78-7.84 (m, 2H), 8.05 (d, J = 7.4 Hz, 1H), 8.21 (d, J = 7.4 Hz, 1H), 9.18 (s, 1H); ^{13}C NMR (100 MHz, DMSO-D_6): δ 112.5, 116.9, 118.5, 120.6, 121.0, 123.2, 125.7, 128.9, 131.0, 1319, 134.0, 135.6, 146.5, 148.9, 155.0, 156.2, 165.5, 172.4; MS: calcd. for $C_{18}H_9F_3N_2O_4Na^+$ 397.0407, found 397. 0392.

(E)-2-(4-(2-(6,8-dichloro-2-phenyl-2H-chromen-3-yl)vinyl)phenyl)-4,4,5,5-tetramethyl-1,3,2-dioxaborolane 6c:

6c was synthesized using reported protocol.[1]

General procedure for the synthesis of compounds 2a-i and 4a-c:

In a 20 mL oven dried sealed tube CuCl (10 mol%), ligand (10 mol%), NaO*t*Bu (20 mol%), $B_2(pin)_2$ (1.2 eq.) were added followed by addition of coumarins/chromenes **1a-i/3a-c** (0.5 mmol) and MeOH (2 eq.) in required amounts under nitrogen environment. In the reaction mixture, solvent (1.5 mL) was added and the reaction was stirred at room temperature for 16h. The progress of reaction was monitored by TLC and on completion the reaction, mixture was diluted by 5 mL ethyl acetate and filtered through a small bed of celite. The filtrate was concentrated and recrystallized with hexane.

Note: Purification of these compounds could not be performed by column chromatography due to their unstable nature.

Ethyl 2-oxo-4-(4,4,5,5-tetramethyl-1,3,2-dioxaborolan-2-yl)chromane-3-carboxylate 2a:
^{1}H NMR (400 MHz, $CDCl_3$): δ 1.19-1.27 (m, 15H), 3.49 (s, 1H), 3.76 (s, 1H), 4.13-4.25 (m, 2H), 6.79-6.86 (m, 2H), 7.07 (d, J = 7.6 Hz, 2H); ^{13}C NMR (100 MHz, $CDCl_3$): δ 24.5, 31.6, 52.6, 61.7, 84.4, 117.7, 121.0, 124.0, 128.0, 130.0, 154.6, 169.7, 170.2; MS: calcd. for $C_{18}H_{23}BO_6H^+$ 347.1660, found 347.0892.

Methyl 2-oxo-4-(4,4,5,5-tetramethyl-1,3,2-dioxaborolan-2-yl)chromane-3-carboxylate 2b:
^{1}H NMR (400 MHz, $CDCl_3$): δ 1.27 (s, 12H), 3.52 (s, 3H), 3.78 (s, 3H), 4.11-4.19 (m, 2H), 6.81-6.89 (m, 2H), 7.07-7.12 (m, 2H); ^{13}C NMR (100 MHz, $CDCl_3$): δ 24.5, 31.6, 52.5, 60.4, 83.2, 118.0, 121.2, 124.0, 128.1, 130.1, 154.5, 170.1; MS: calcd. for $C_{17}H_{21}BO_6Na^+$ 355.1323, found 355.0985.

4-(4,4,5,5-tetramethyl-1,3,2-dioxaborolan-2-yl)chroman-2-one 2c:
^{1}H NMR (400 MHz, $CDCl_3$): δ 1.27 (s, 12H), 3.40 (s, 2H), 3.47-3.54 (m, 1H), 6.65-6.71 (m, 2H), 6.74-6.87 (m, 1H), 7.01 (d, J = 7.2 Hz, 1H); ^{13}C NMR (100 MHz, $CDCl_3$): δ 24.6, 31.6, 51.8, 83.2, 118.5, 124.1, 128.2, 174.4.

2-oxo-4-(4,4,5,5-tetramethyl-1,3,2-dioxaborolan-2-yl)chromane-3-carbonitrile 2d:
^{1}H NMR (400 MHz, $CDCl_3$): δ 1.27 (s, 12H), 3.61 (d, J = 6 Hz, 1H), 3.83 (s, 1H), 6.77-6.90 (m, 2H), 7.10-7.16 (m, 2H); ^{13}C NMR (100 MHz, $CDCl_3$): δ 24.7, 31.6, 53.6, 83.3, 117.6, 119.9, 120.9, 128.8, 131.0, 133.8, 137.0, 161.6, 167.6

3-acetyl-4-(4,4,5,5-tetramethyl-1,3,2-dioxaborolan-2-yl)chroman-2-one 2e:
^{1}H NMR (400 MHz, $CDCl_3$): δ 1.16 (s, 12H), 2.5 (s, 3H), 2.75 (d, J = 5.8 Hz, 1H), 3.86 (d, J = 5.8 Hz, 1H), 7.05-7.12 (m, 2H), 7.18-7.24 (m, 1H), 7.31-7.33 (m, 1H); ^{13}C NMR (100 MHz, $CDCl_3$): δ 24.5, 30.5, 31.6, 53.8, 83.6, 116.7, 123.3, 124.7, 127.7, 150.8, 165.6, 202.6; MS: calcd. for $C_{17}H_{21}BO_5Na^+$ 339.1374, found 339.0927.

Methyl 6-chloro-2-oxo-4-(4,4,5,5-tetramethyl-1,3,2-dioxaborolan-2-yl)chromane-3-carboxylate 2f:

^{1}H NMR (400 MHz, $CDCl_3$): δ 1.12 (s, 12H), 3.48 (s, 3H), 3.68 (s, 3H), 4.01-4.08 (m, 2H), 6.70 (d, J = 8.4 Hz, 1H), 6.94-6.99 (m, 2H); ^{13}C NMR (100 MHz, $CDCl_3$): δ 24.5, 31.6, 53.0, 60.4, 83.1, 118.2, 125.4, 128.0, 170.0; MS: calcd. for $C_{17}H_{20}BClO_6Na^+$ 389.0934, found 389.0843.

Methyl 6,8-dichloro-2-oxo-4-(4,4,5,5-tetramethyl-1,3,2-dioxaborolan-2-yl)chromane-3-carboxylate 2g:

^{1}H NMR (400 MHz, $CDCl_3$): δ 1.25 (s, 12H), 3.56 (s, 3H), 3.77 (s, 3H), 4.06-4.15 (m, 2H), 7.08 (d, J = 1.6 Hz, 1H), 7.20 (d, J = 1.6 Hz, 1H); ^{13}C NMR (100 MHz, $CDCl_3$): δ 24.7, 31.6, 52.5, 60.4, 83.5, 121.5, 125.3, 127.3, 129.5, 148.7, 169.5, MS: calcd. for $C_{17}H_{19}BCl_2O_6Na^+$ 423.0544, found 423.0300.

3-amino-4-(4,4,5,5-tetramethyl-1,3,2-dioxaborolan-2-yl)chroman-2-one 2h:

^{1}H NMR (400 MHz, $CDCl_3$): δ 1.25 (s, 12H), 2.26 (s, 1H), 3.61 (s, 3H), 5.51 (s, 2H), 6.64-6.81 (m, 2H), 6.7.02-7.22 (m, 2H); ^{13}C NMR (100 MHz, $CDCl_3$): δ 24.7, 31.6, 52.6, 60.4, 82.8, 116.8, 123.3, 125.1, 127.8, 128.4, 150.14. 168.5, 170.2.

2-(4,4,5,5-tetramethyl-1,3,2-dioxaborolan-2-yl)chroman-4-one 4a:

^{1}H NMR (400 MHz, $CDCl_3$): δ 1.32 (s, 12H), 2.72-2.77 (m, 1H), 2.88-2.96 (m, 1H), 5.51 (s, 2H), 4.47-4.51 (m, 1H), 6.99-7.02 (m, 1H), 7.08 (d, J = 8.2 Hz, 1H), 7.44-7.48 (m, 1H), 7.90 (d, J = 8.2 Hz, 1H); ^{13}C NMR (100 MHz, $CDCl_3$): δ 24.6, 31.6, 38.9, 84.9, 118.3, 121.2, 127.1, 135.7, 162.9, 192.1; MS: calcd. for $C_{15}H_{19}BO_4Na^+$ 297.1269, found 297.0502.

Ethyl 4-(5,5-dimethyl-1,3,2-dioxaborinan-2-yl)-2-oxochromane-3-carboxylate 4b:

^{1}H NMR (400 MHz, $CDCl_3$): δ 1.00 (s, 6H), 1.28-1.29 (m, 3H), 3.51 (s, 1H), 3,67 (m, 4H), 3.78 (s, 1H), 4.09-4.26 (m, 2H), 6.83-6.89 (m, 2H), 7.06-7.09 (m, 1H); ^{13}C NMR (100 MHz, $CDCl_3$): δ 14.1, 21.6, 31.8, 52.7, 61.9, 72.7, 117.7, 121.0, 124.8, 127.8, 130.5, 162.9, 170.2; MS: calcd. for $C_{17}H_{21}BO_6Na^+$ 355.1323, found 355.0962.

Procedure for the synthesis of compounds 5a and 5b:

Potassium ethyl 2-oxo-4-(trifluoro-l4-boranyl)chromane-3-carboxylate 5a:

Solid KHF_2 (1.5 mmol) was added to a solution of **2a** (0.25 mmol) in acetonitrile (2 mL) at 0 ºC. Water (2 mL) was added to the stirred suspension at 0 ºC. The mixture was warmed to room temperature then stirred for 3 h. The solvent was evaporated, hot acetone was added, removed the inorganic salts by filtration, the filtrate was dilute with hexane, then stored at fridge overnight. The resulting solid was collected after filtration and dissolved in water then remove water on vacuum. Then afforded product **5a**.
^{1}H NMR (400 MHz, Acetone-d_6): δ 1.20-1.25 (m, 3H), 3.29 (s, 1H), 3.64-3.66 (m, 2H), 4.09-4.16 (m, 1H), 6.43-6.65 (m, 2H), 6.77-6.99 (m, 2H); ^{13}C NMR (100 MHz, Acetone-d_6): δ 15.3, 52.8, 61.9, 75.7, 112.3, 118.0, 120.7, 126.9, 128.6, 135.0, 172.5.

Ethyl 4-hydroxy-2-oxochromane-3-carboxylate 5b:
A 20 mL oven dried sealed tube was charged with **2a** (0.5 mmol), B_2pin_2 (0.6 mmol), CuCl (10 mol%), PCy_3 (10 mol%), NaO*t*Bu (20 mol%) and MeOH (1.0 mmol) followed by addition of THF (1 mL) under nitrogen atmosphere. The reaction was performed at room temperature for 16h. Upon completion, it was filtered over a celite pad and volatiles were removed. Subsequently, $NaBO_3.4H_2O$ (2.0 mmol), water (2.0 mL) and THF (3 mL) were added and the reaction mixture was stirred for 4 hours. Upon completion, the reaction mixture was diluted with 50% NaCl solution, and extracted thrice with EtOAc. The combined organic fractions were washed five times with water to remove pinacol and once with brine and dried over $MgSO_4$.

General procedure for the synthesis of compounds 7a-c:
In a 20 mL oven dried sealed tube CuCl (10 mol%), ligand (10 mol%), NaO*t*Bu (20 mol%), $B_2(pin)_2$ (1.2 eq.) were added followed by addition of coumarins/chromenes **6a-c** (0.5 mmol) and MeOH (2 eq.) in required amounts under nitrogen atmosphere. In the reaction mixture solvent (1.5 mL) was added and the reaction was stirred at room temperature for 16h. The progress of reaction was monitored by TLC and on completion the reaction, mixture was diluted by 5 mL ethyl acetate and filtered through a small bed of celite. The filtrate was concentrated and recrystallized with hexane.
Note: Purification of these compounds could not be performed by column chromatography due to their unstable nature.

6,8-dichloro-2-phenyl-4-(4,4,5,5-tetramethyl-1,3,2-dioxaborolan-2-yl)chromane-3-carbonitrile 7a:

^{1}H NMR (400 MHz, $CDCl_3$): δ 1.22 (s, 12H), 2.60-2.62 (m, 1H), 3.71-3.75 (m, 1H), 7.04 (s, 1H), 7.11-7.15 (m, 1H), 7.26-7.34 (m, 4H), 7.39-7.43 (m, 2H), 7.72 (s, 1H); ^{13}C NMR (100 MHz, $CDCl_3$): δ 24.5, 31.6, 84.5, 108.3, 118.5, 120.8, 127.0, 127.8, 128.8, 130.3, 133.4, 145.0, 148.7.

4-(4,4,5,5-tetramethyl-1,3,2-dioxaborolan-2-yl)-3-(3-(2-(trifluoromethoxy)phenyl)-1,2,4-oxadiazol-5-yl)chroman-2-one 7b:

^{1}H NMR (400 MHz, DMSO-d_6): δ 1.16 (s, 12H), 3.43 (s, 1H), 3.71 (s, 1H), 7.39 (s, 4H), 7.45-7.47 (m, 1H), 7.56-7.65 (m, 2H), 7.95 (s, 1H); ^{13}C NMR (100 MHz, DMSO-d_6): δ 25.2 (s, 12H), 31.4, 52.7, 81.8, 123.1, 127.9, 128.8, 129.2, 130.6, 131.8, 133.2, 146..4, 165.1; ^{19}F NMR (376 MHz, DMSO-d_6): δ -56.4.

2-(4-(2-(6,8-dichloro-2-phenyl-2*H*-chromen-3-yl)-2-(4,4,5,5-tetramethyl-1,3,2-dioxaborolan-2-yl)ethyl)phenyl)-4,4,5,5-tetramethyl-1,3,2-dioxaborolane 7c:

^{1}H NMR (400 MHz, $CDCl_3$): δ 1.24 (s, 12H), 1.33 (s, 12H), 2.37-2.51 (m, 2H), 5.84 (s, 1H), 6.25 (s, 1H), 6.79 (s, 1H), 7.03 (s, 1H), 7.16-7.29 (m, 7H), 7.67 (d, J = 7.6 Hz, 2H); ^{13}C NMR (100 MHz, $CDCl_3$): δ 24.9, 35.0, 80.6, 83.7, 118.4, 123.9, 127.6, 128.8, 135.0, 133.4; $C_{35}H_{40}B_2Cl_2O5Na^+$ 655.2331, found 355.4053.

References:

1. Y. Zhong, Y. Wu, R. Liu, Z. Li, Y. Chen, T. Evans, P. Chuang, B. Das and J. C. He, *PLoS One*, 2011, **6**, e27945

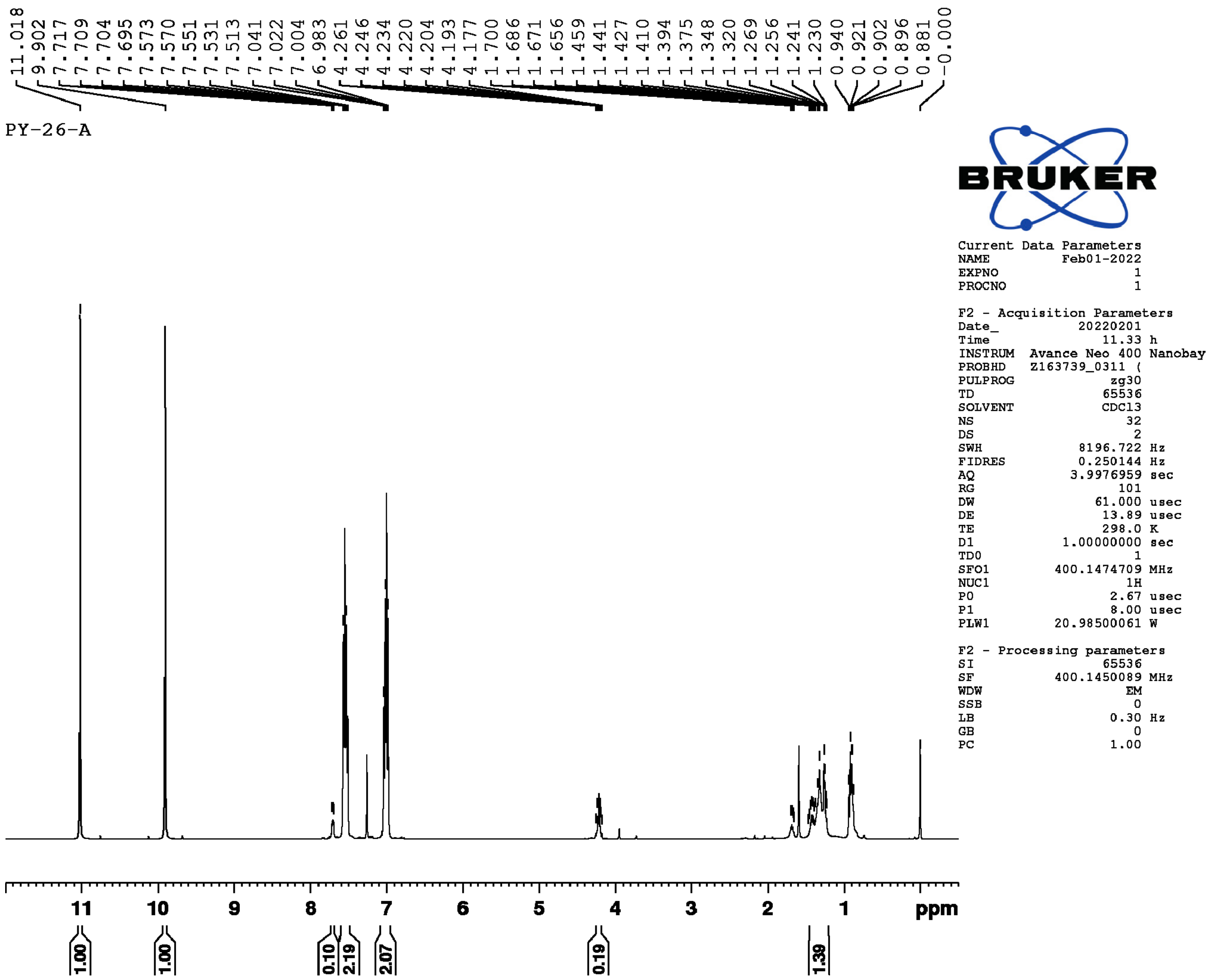

Figure 1. Formation of salicylaldehyde **2a'** (^{1}H NMR spectrum of isolated salicylaldehyde)

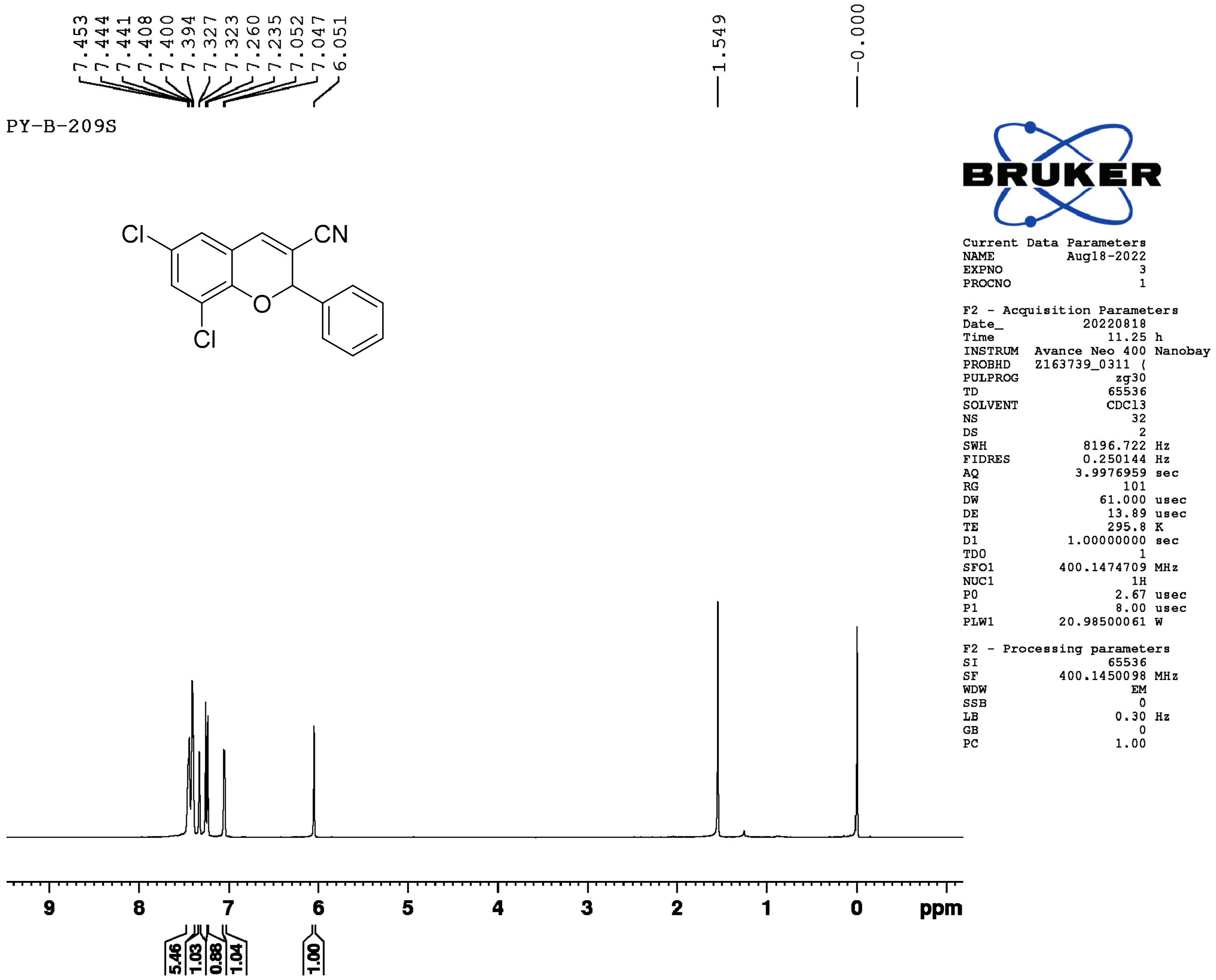

^{1}H NMR spectrum of **6a**

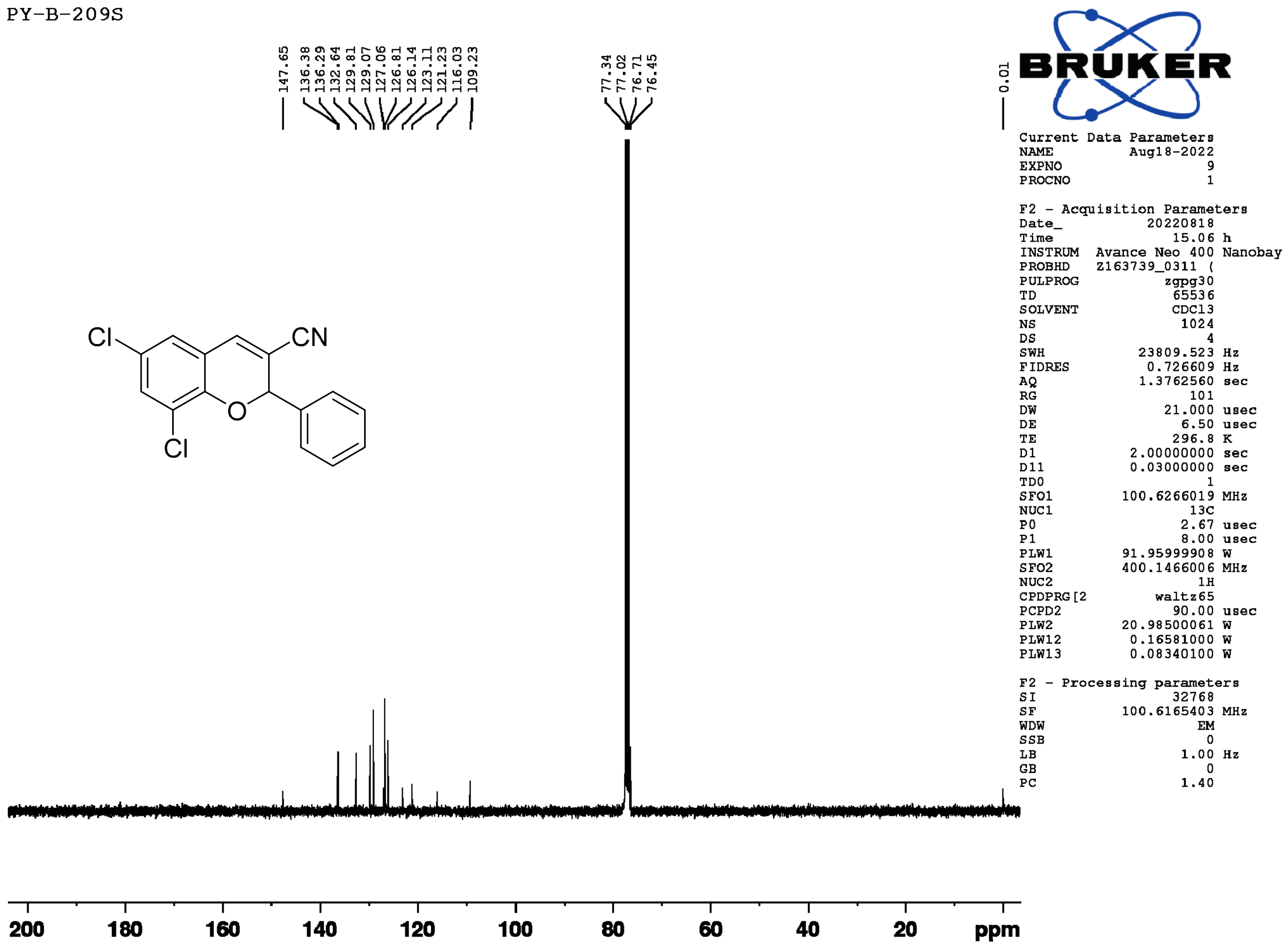

^{13}C NMR spectrum of **6a**

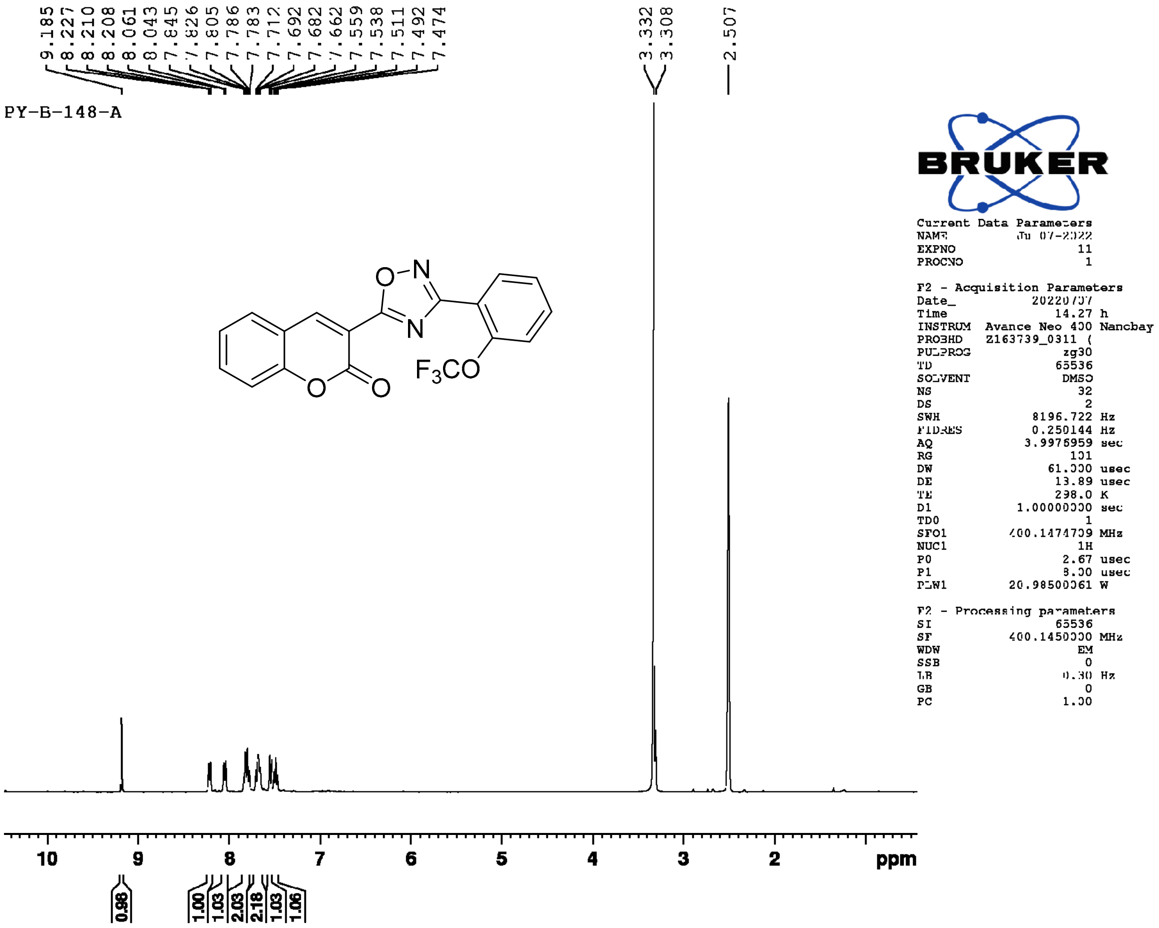

^{1}H NMR spectrum of **6b**

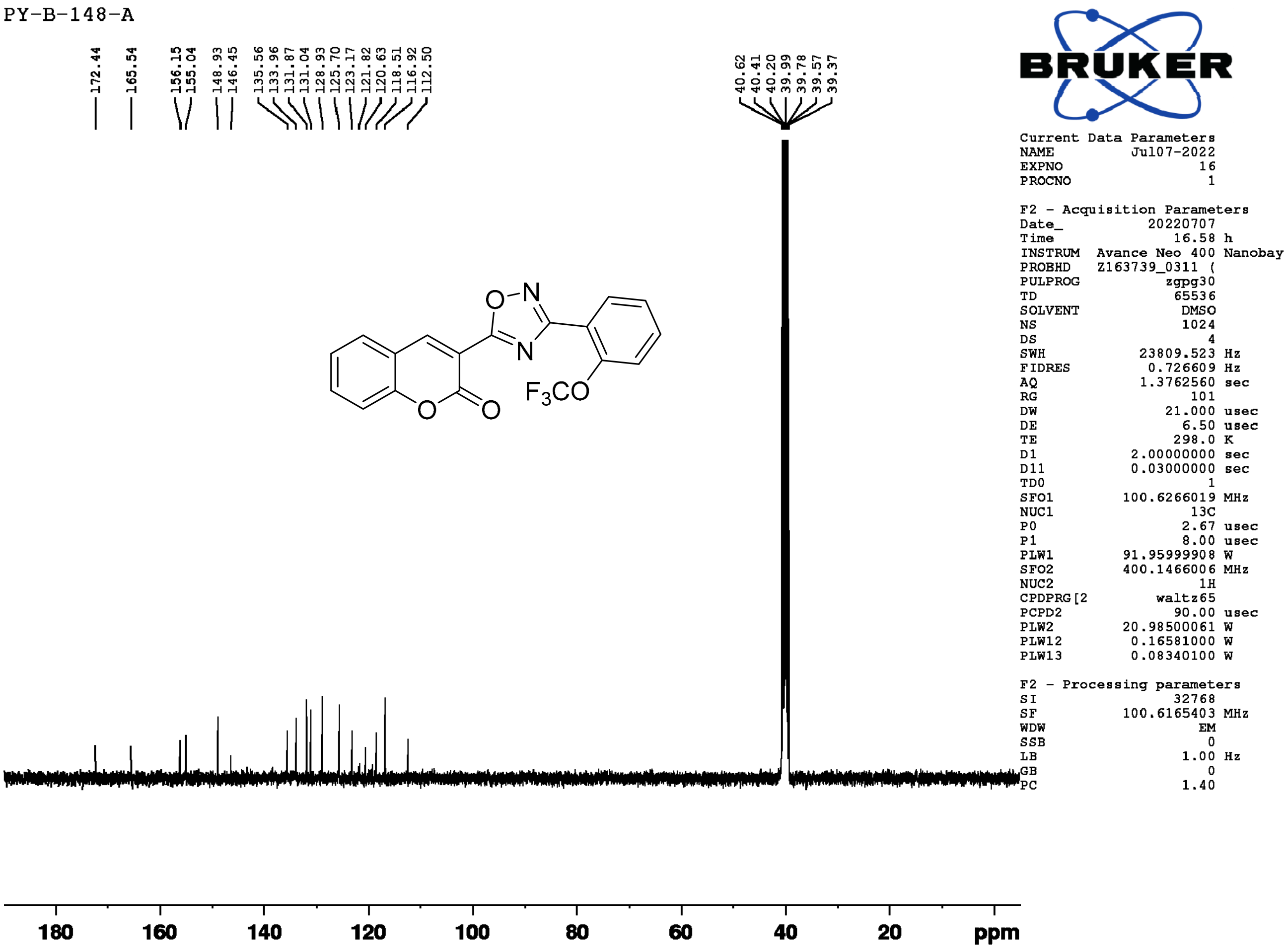

^{13}C NMR spectrum of **6b**

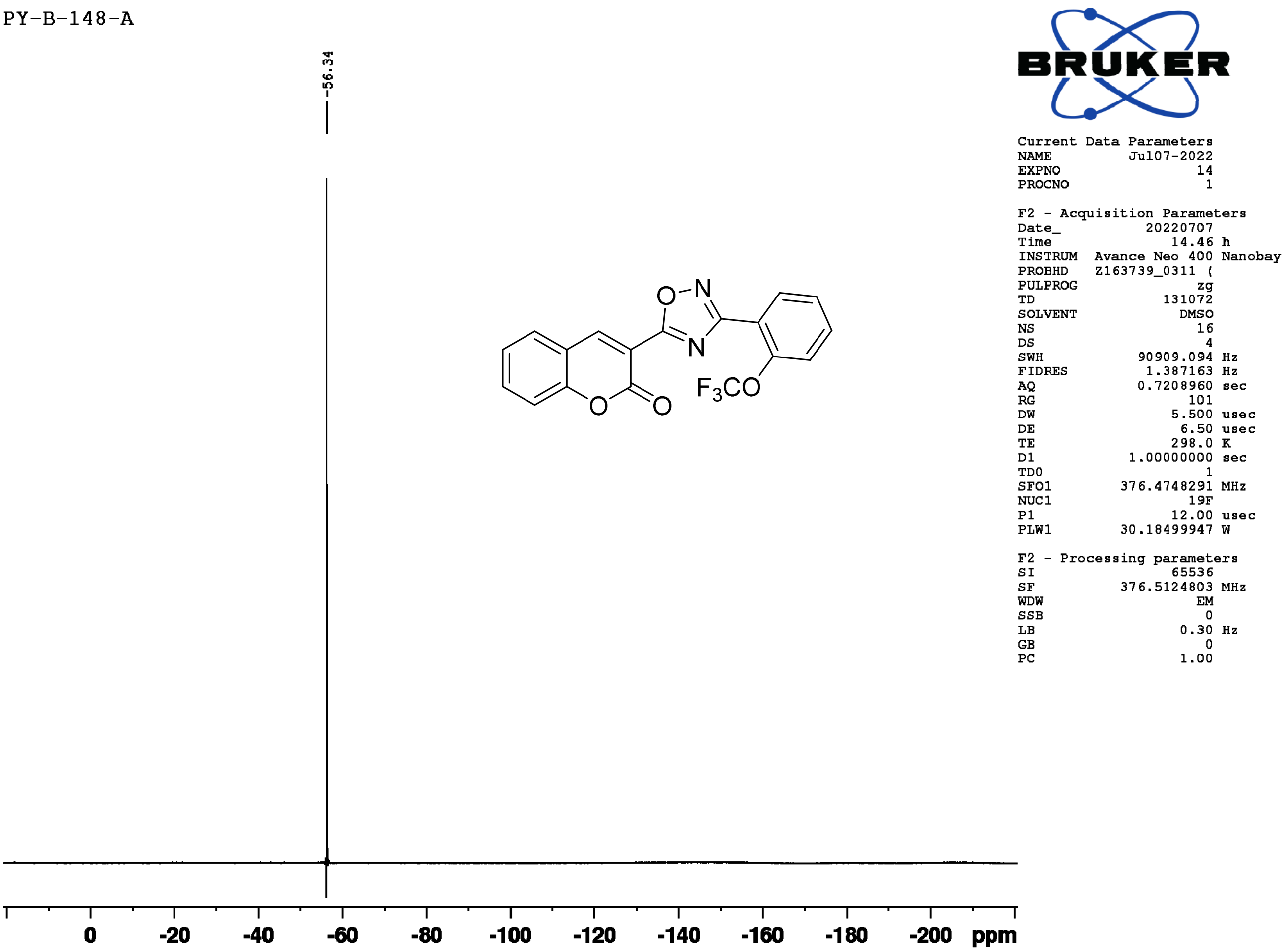

^{19}F NMR spectrum of **6b**

Spectrum View - 6b.d

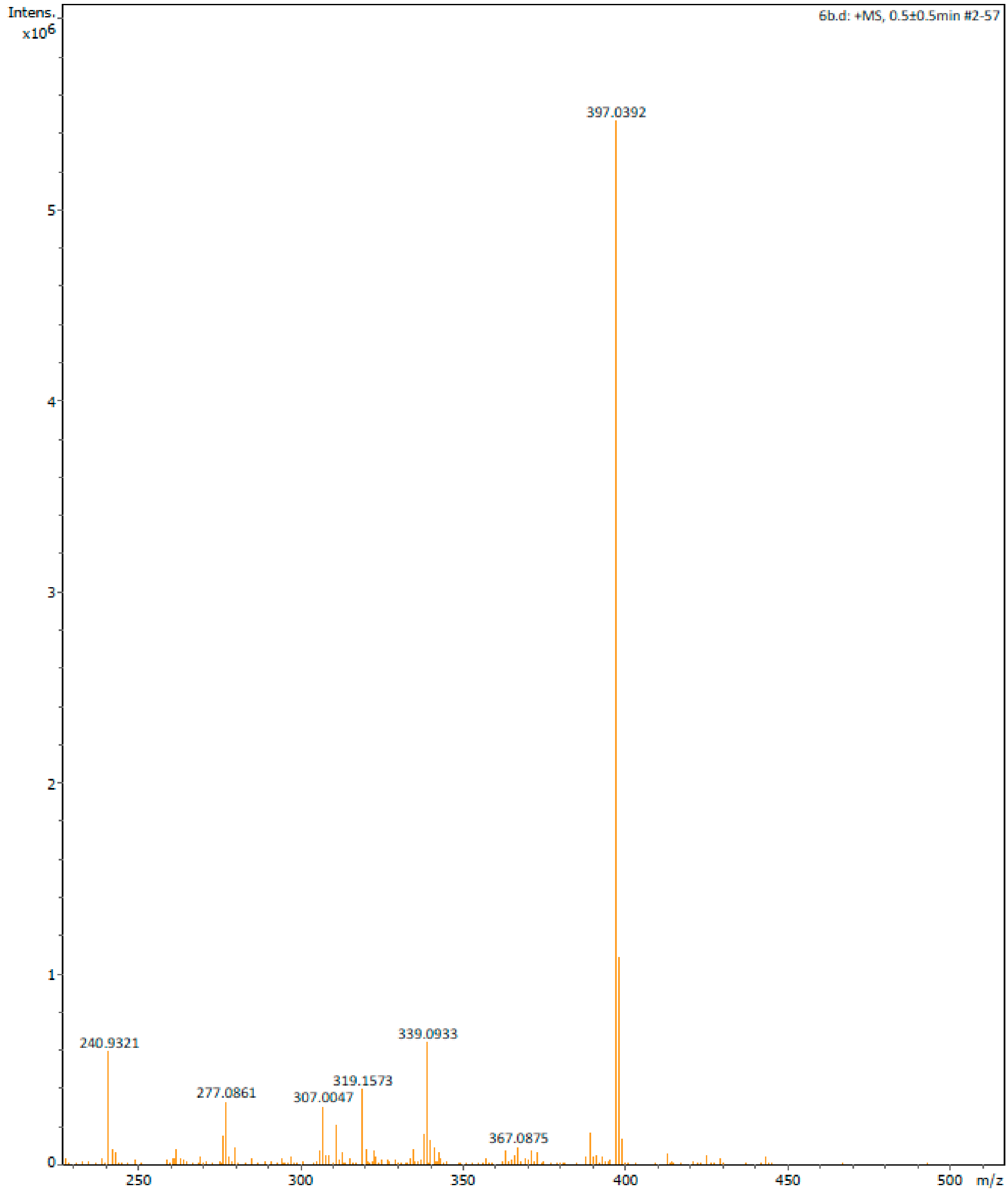

Bruker Compass DataAnalysis 5.2 printed: 9/9/2022 3:45:56 PM by: demo Page 1 of 1

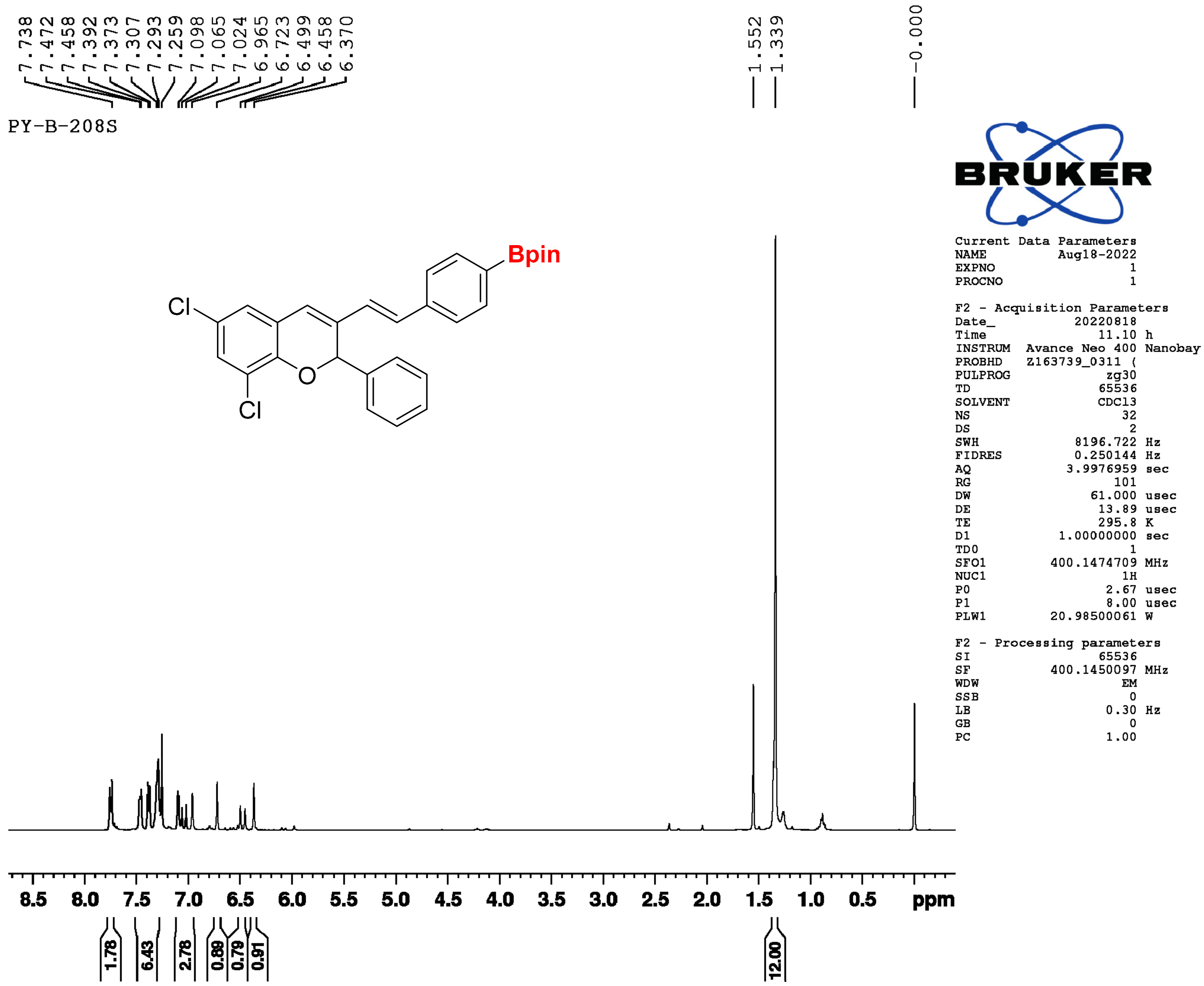

^{1}H NMR spectrum of **6c**

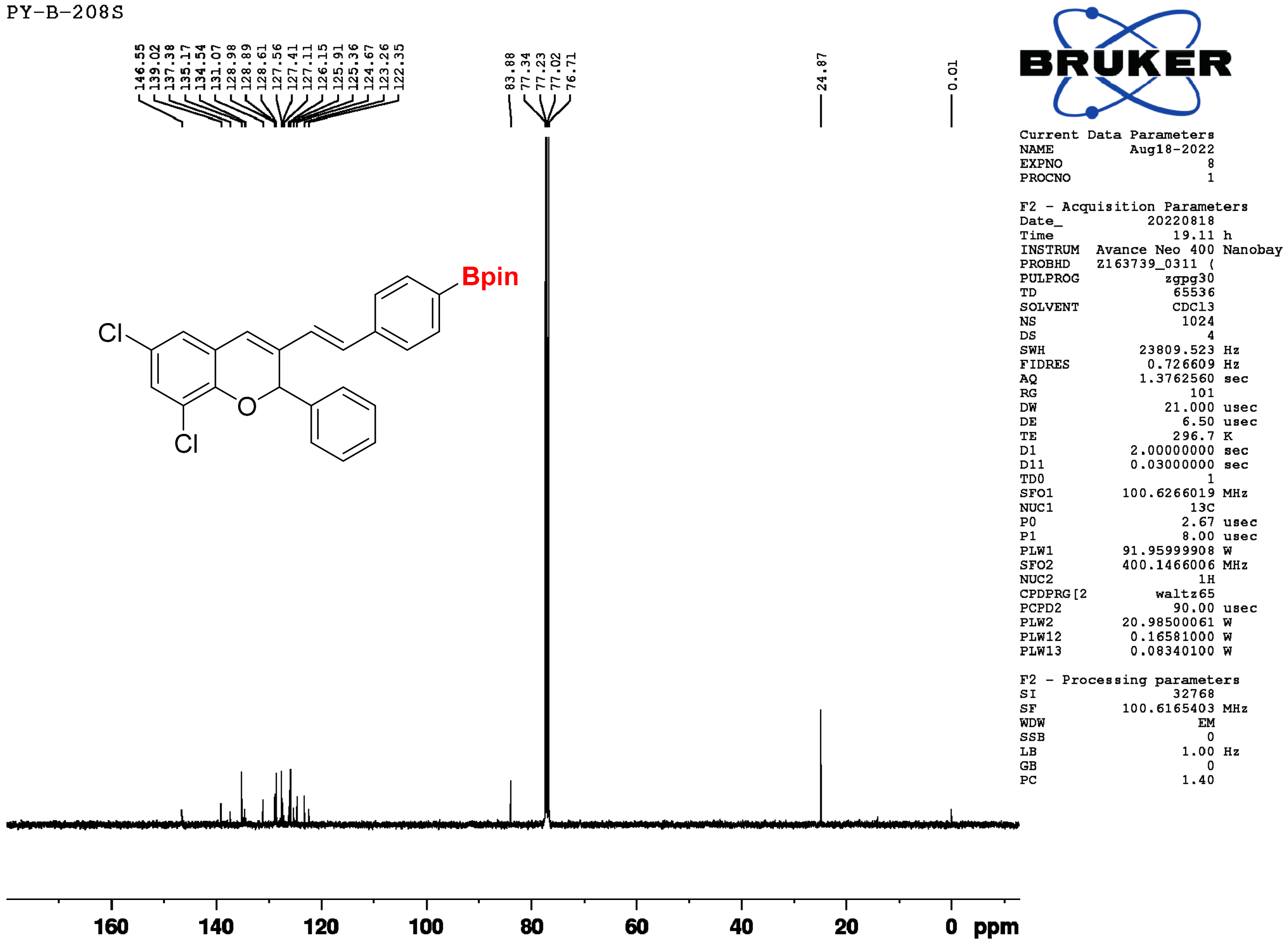

^{13}C NMR spectrum of **6c**

Spectrum View - 6c.d

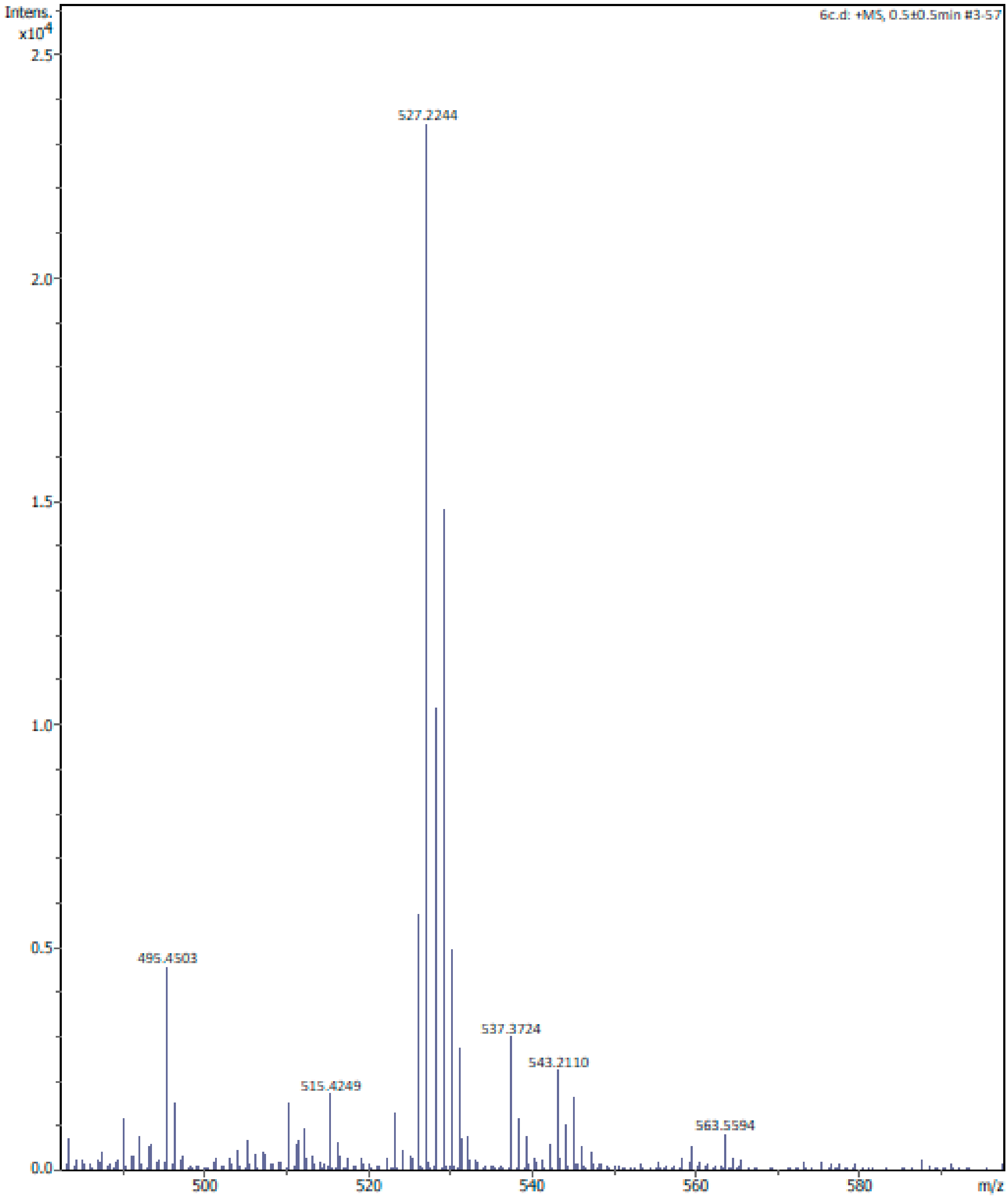

Bruker Compass DataAnalysis 5.2 printed: 9/9/2022 3:53:49 PM by: demo Page 1 of 1

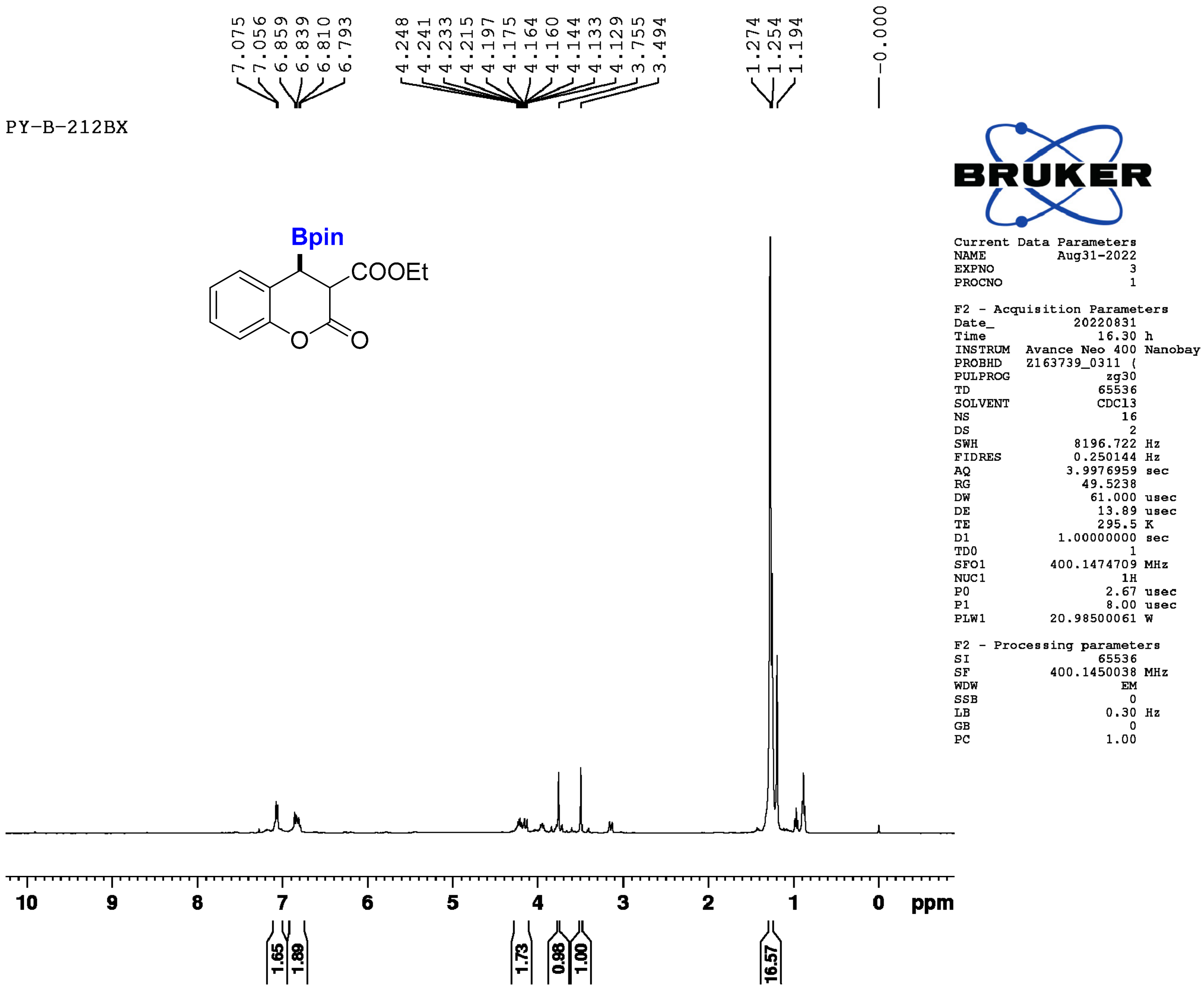

^{1}H NMR spectrum of **2a**

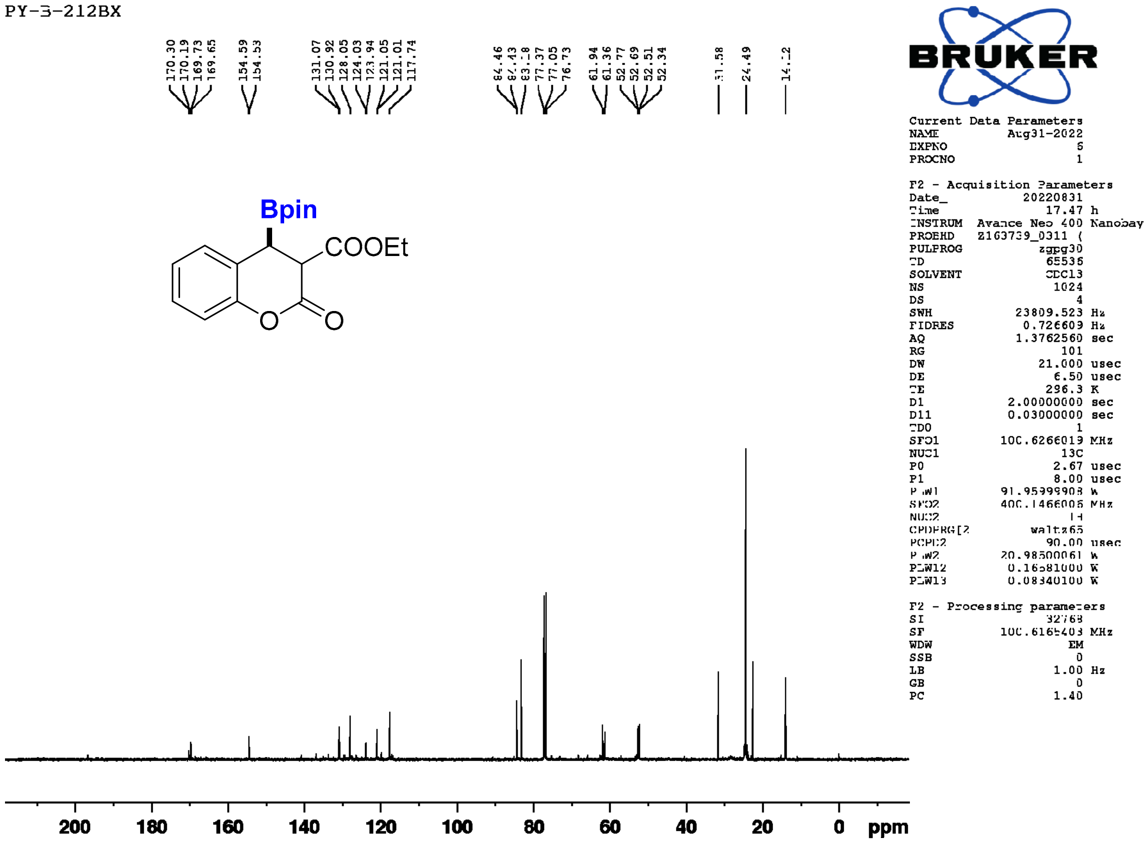

^{13}C NMR spectrum of **2a**

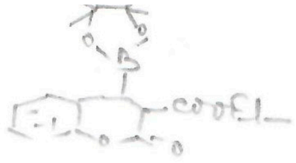

Window Display Report

Compound Spectra - PY-B-212BX1.d

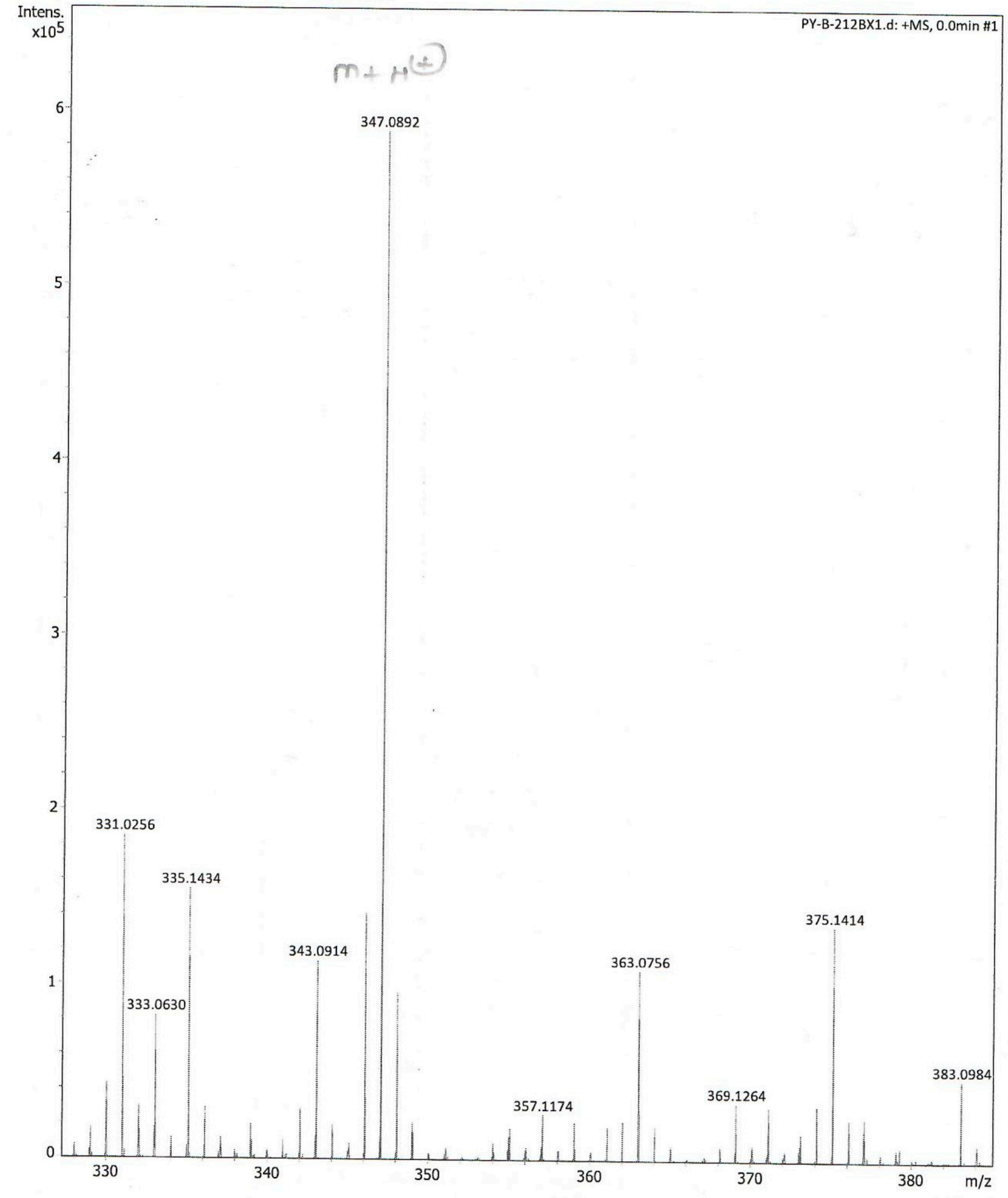

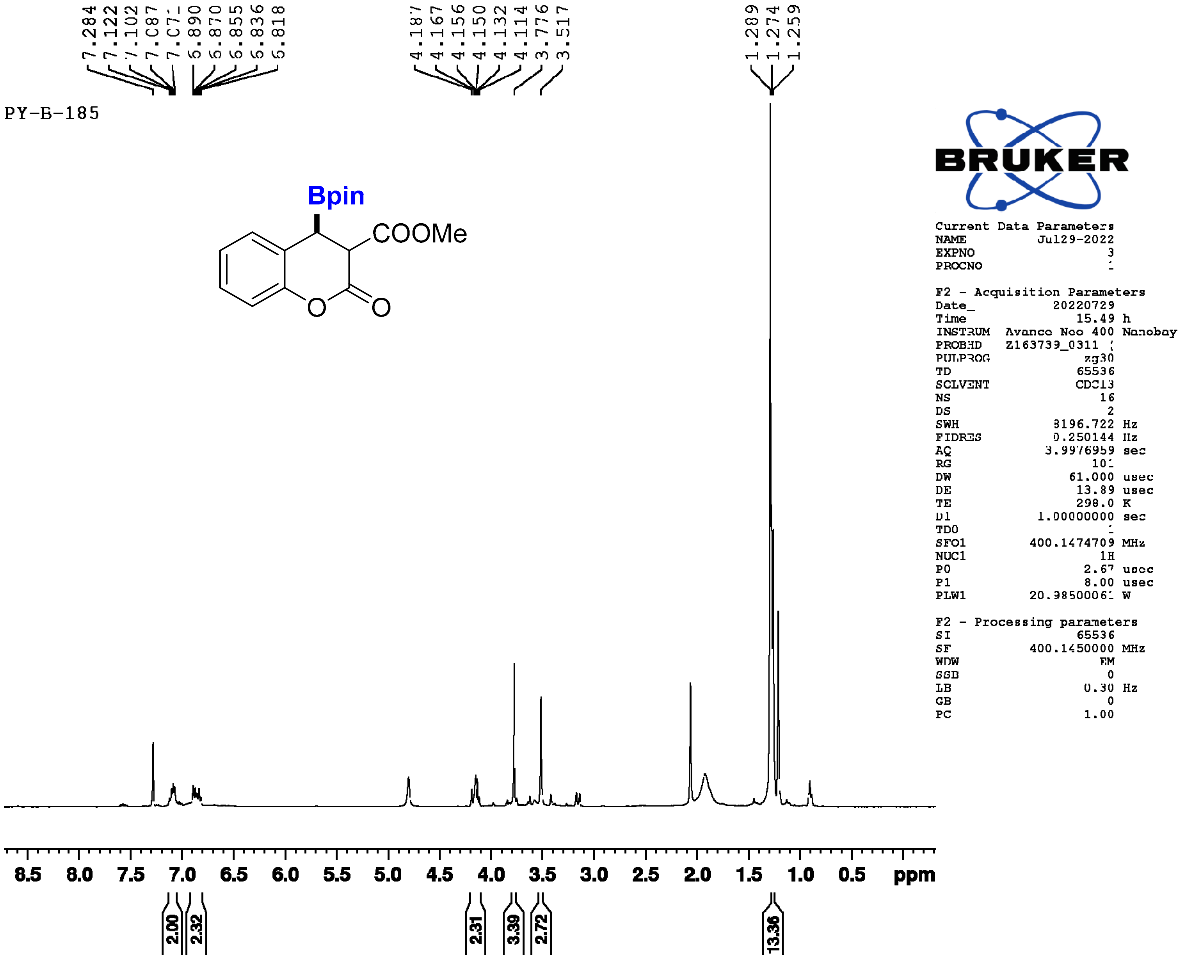

^{1}H NMR spectrum of **2b**

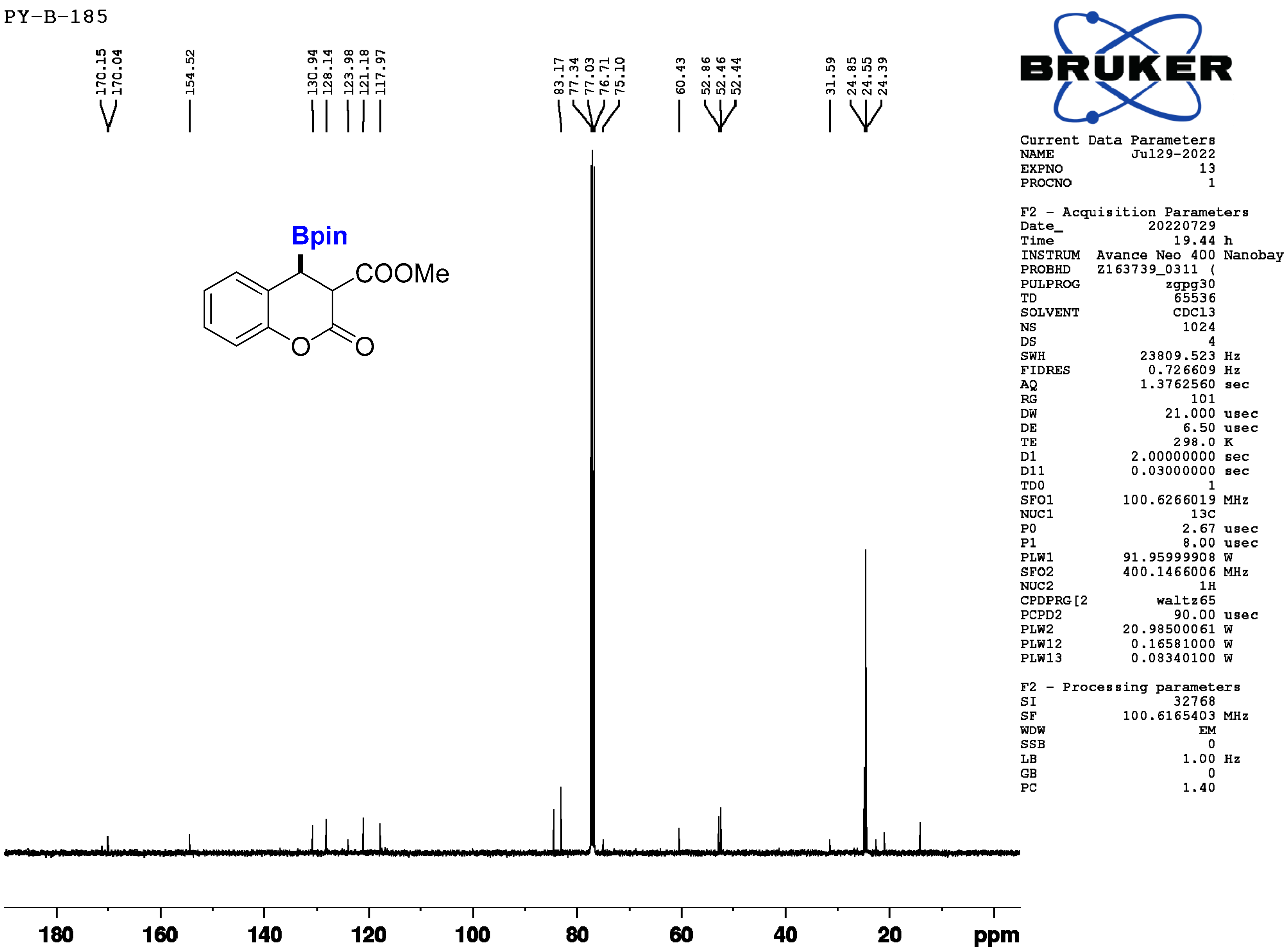

^{13}C NMR spectrum of **2b**

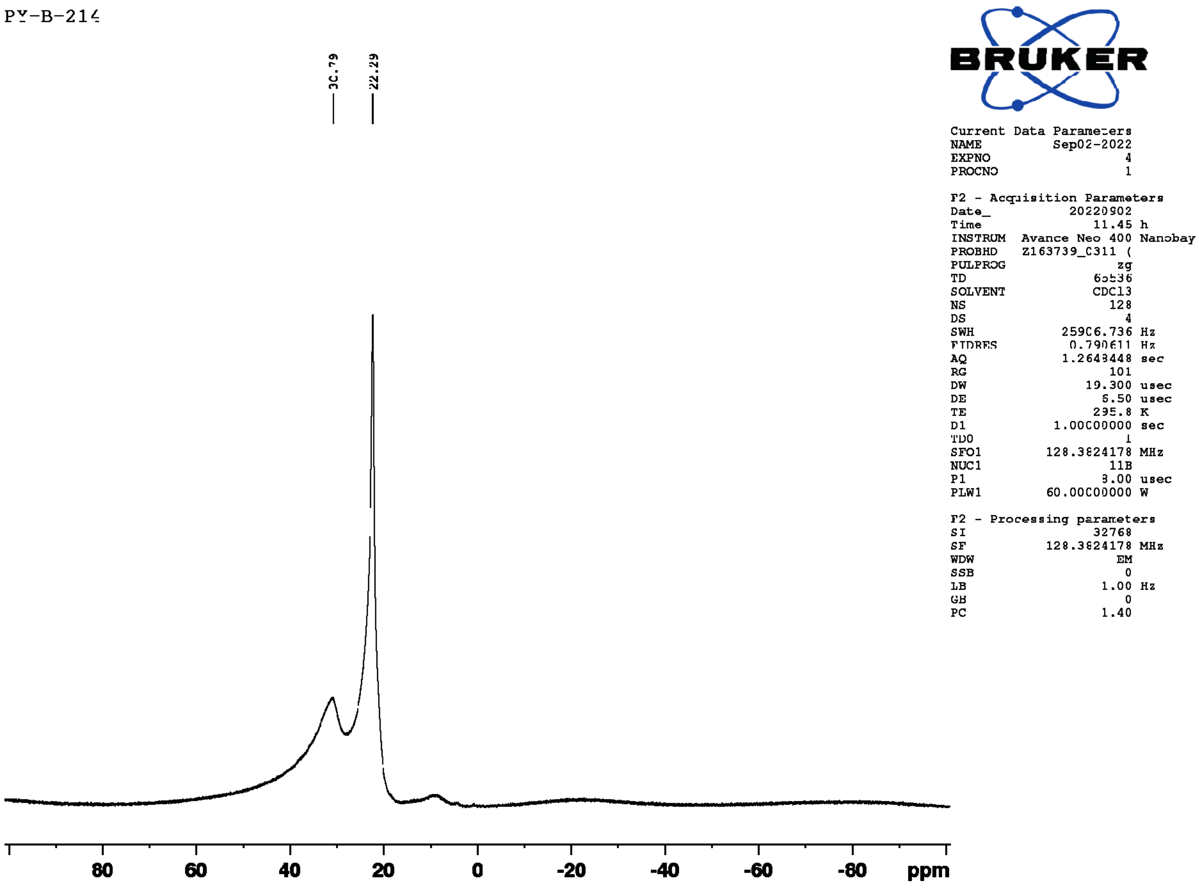

^{11}B NMR spectrum of **2b**

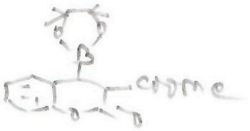

Window Display Report

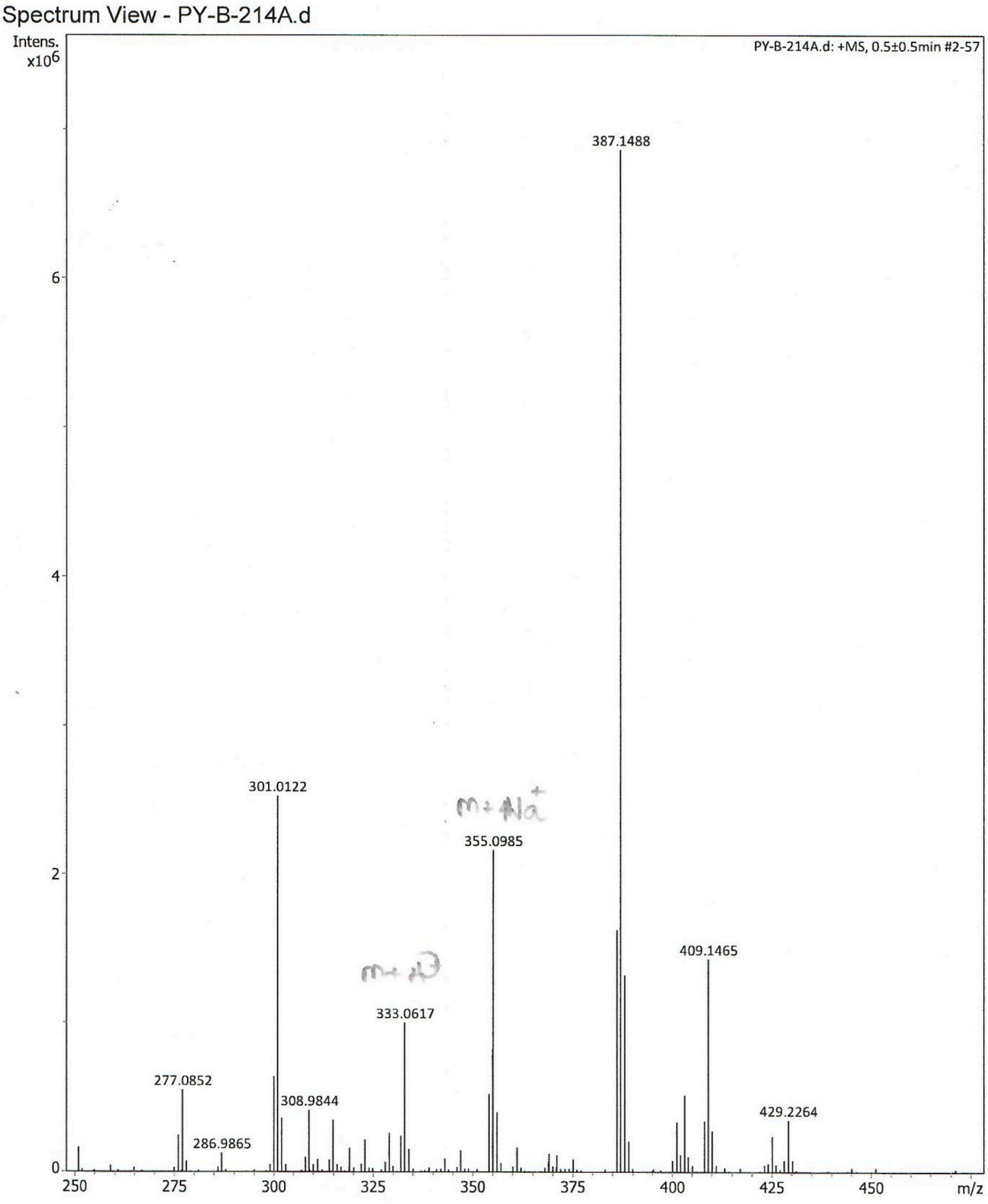

Bruker Compass DataAnalysis 5.2 printed: 9/2/2022 4:10:13 PM by: demo Page 1 of 1

Mass spectrum of 2b

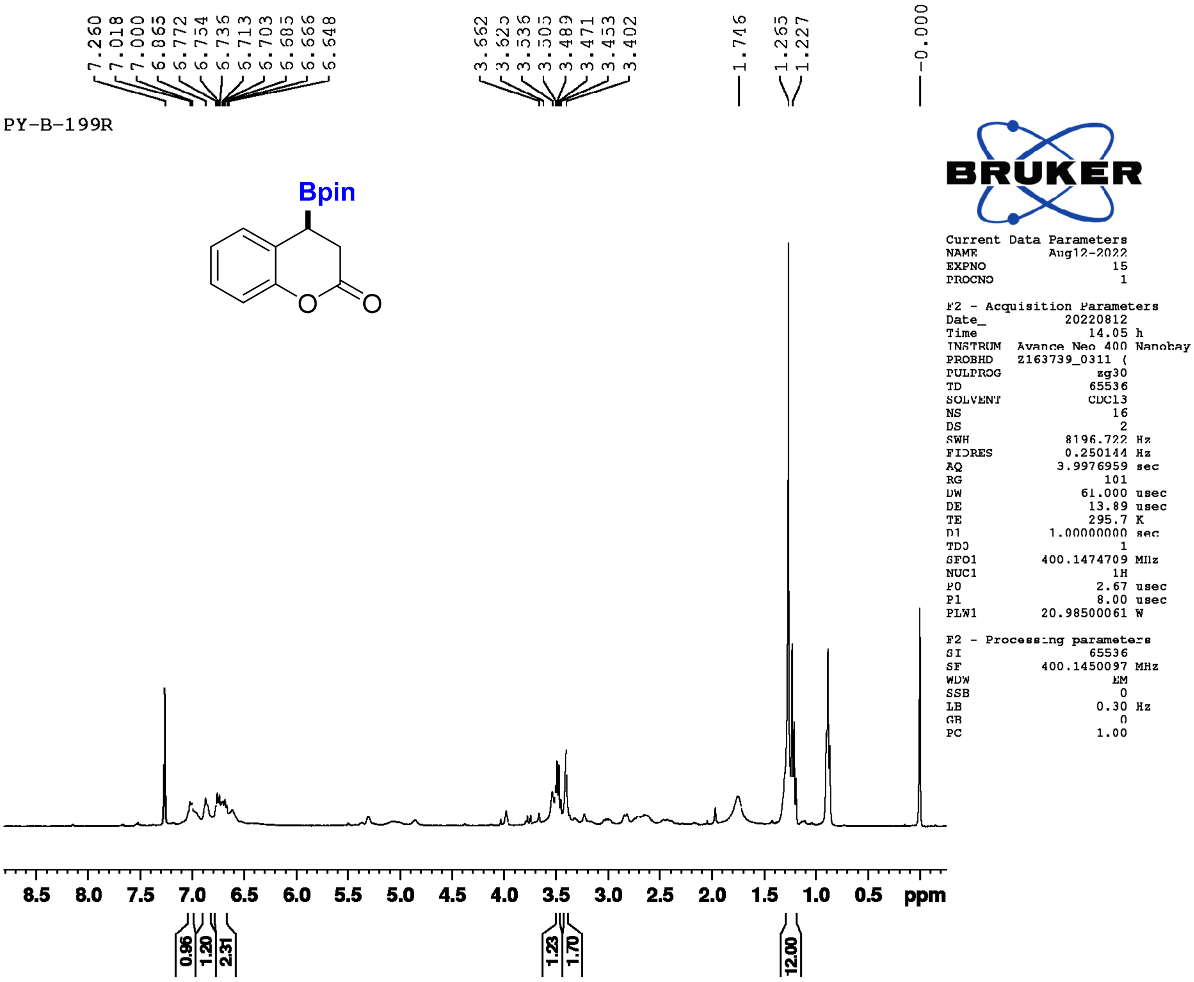

^{1}H NMR spectrum of **2c**

PY-B-199R

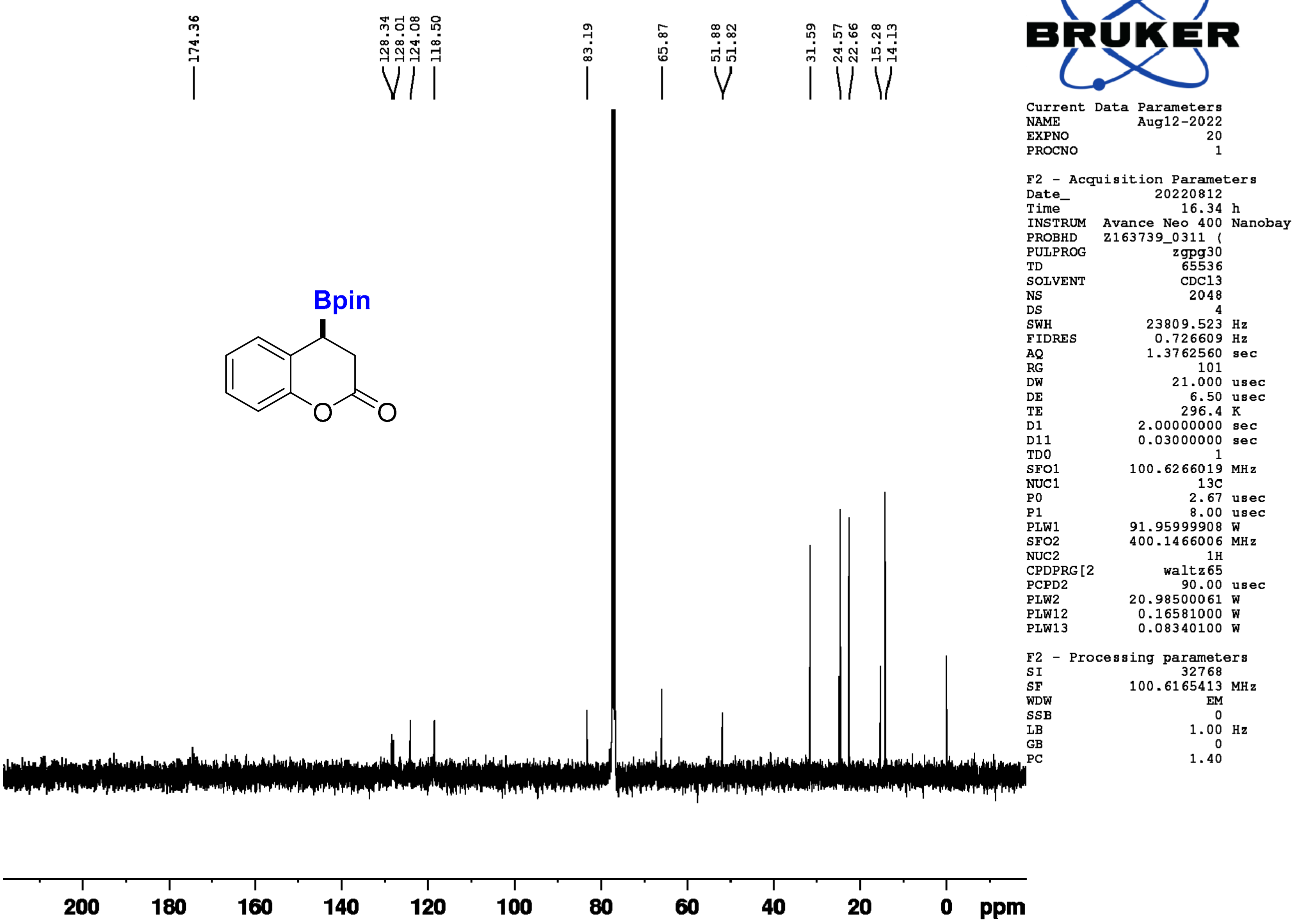

^{13}C NMR spectrum of **2c**

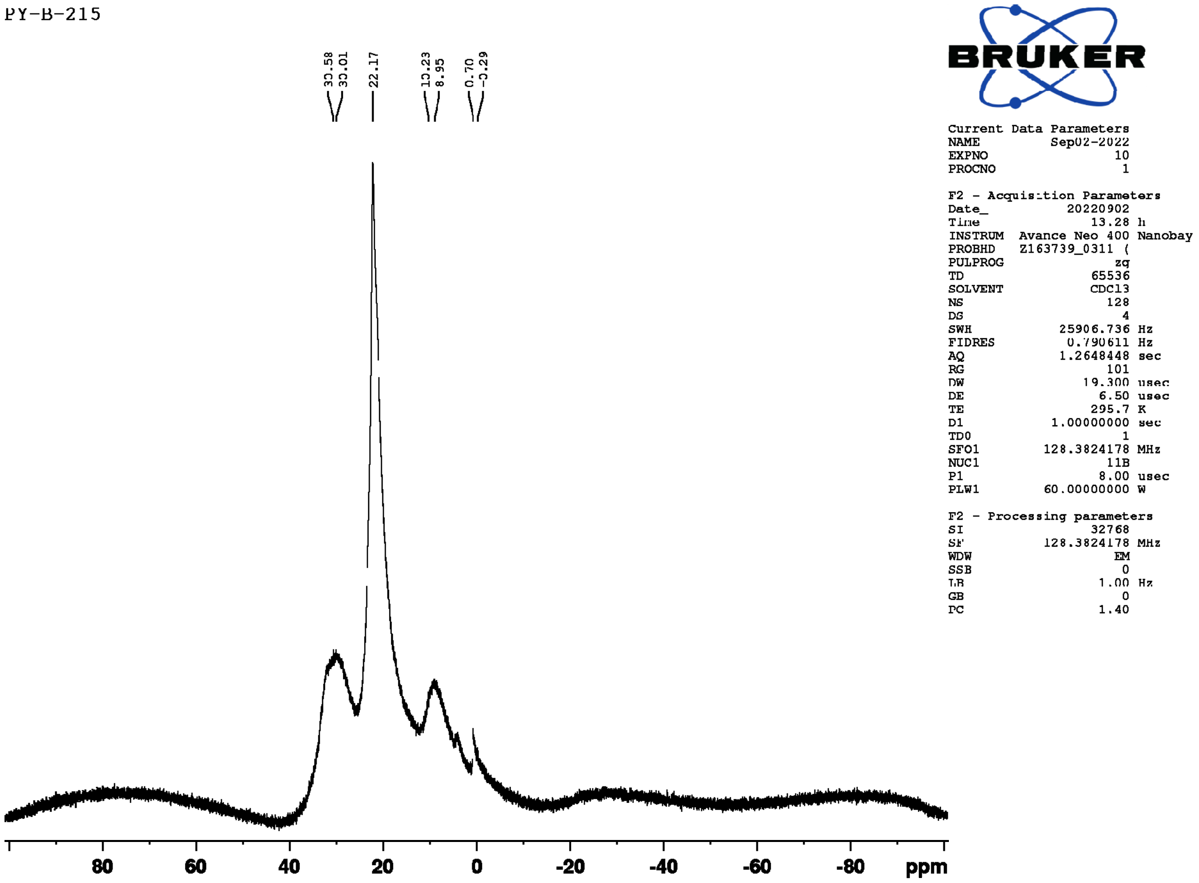

^{11}B NMR spectrum of **2c**

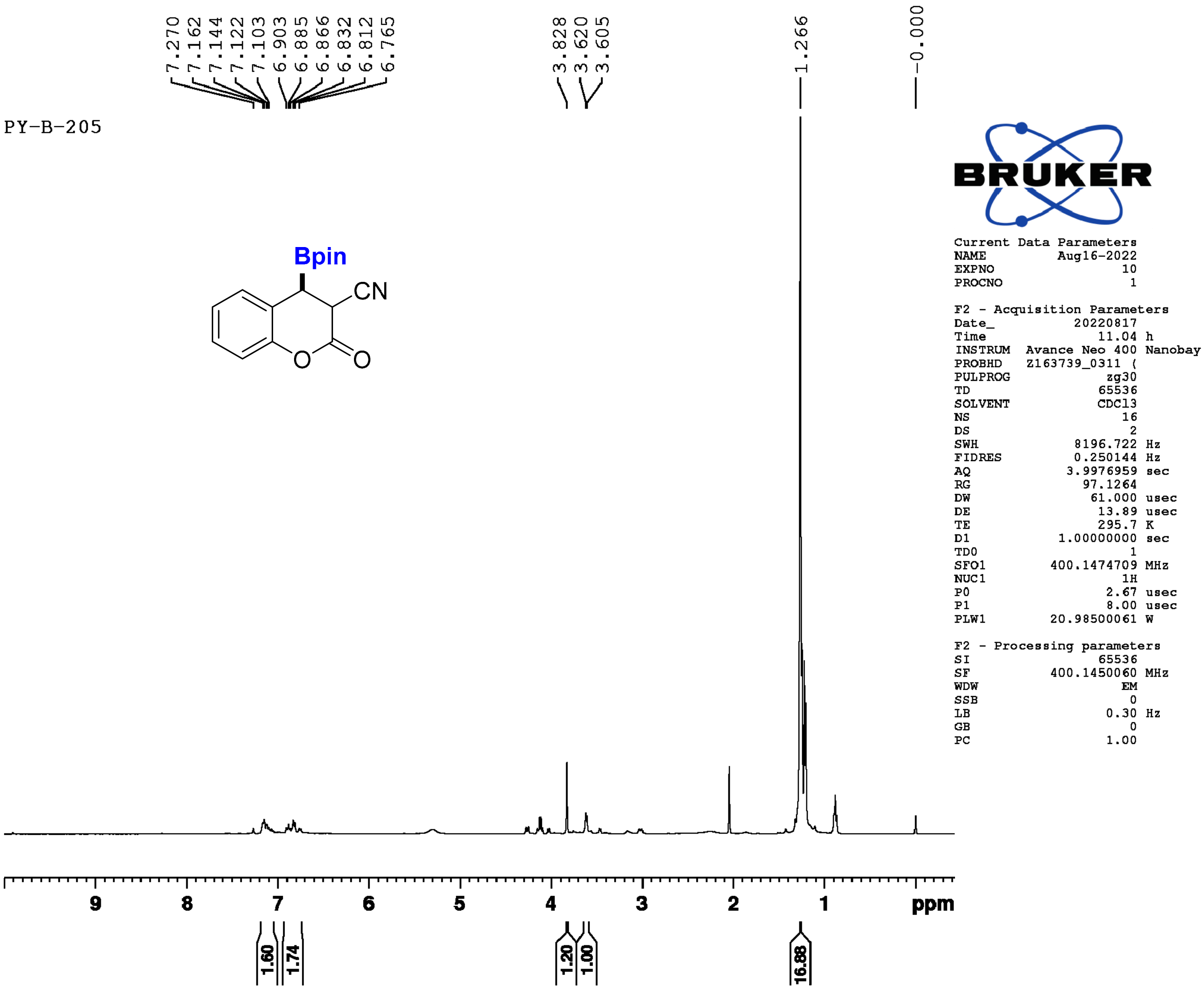

^{1}H NMR spectrum of **2d**

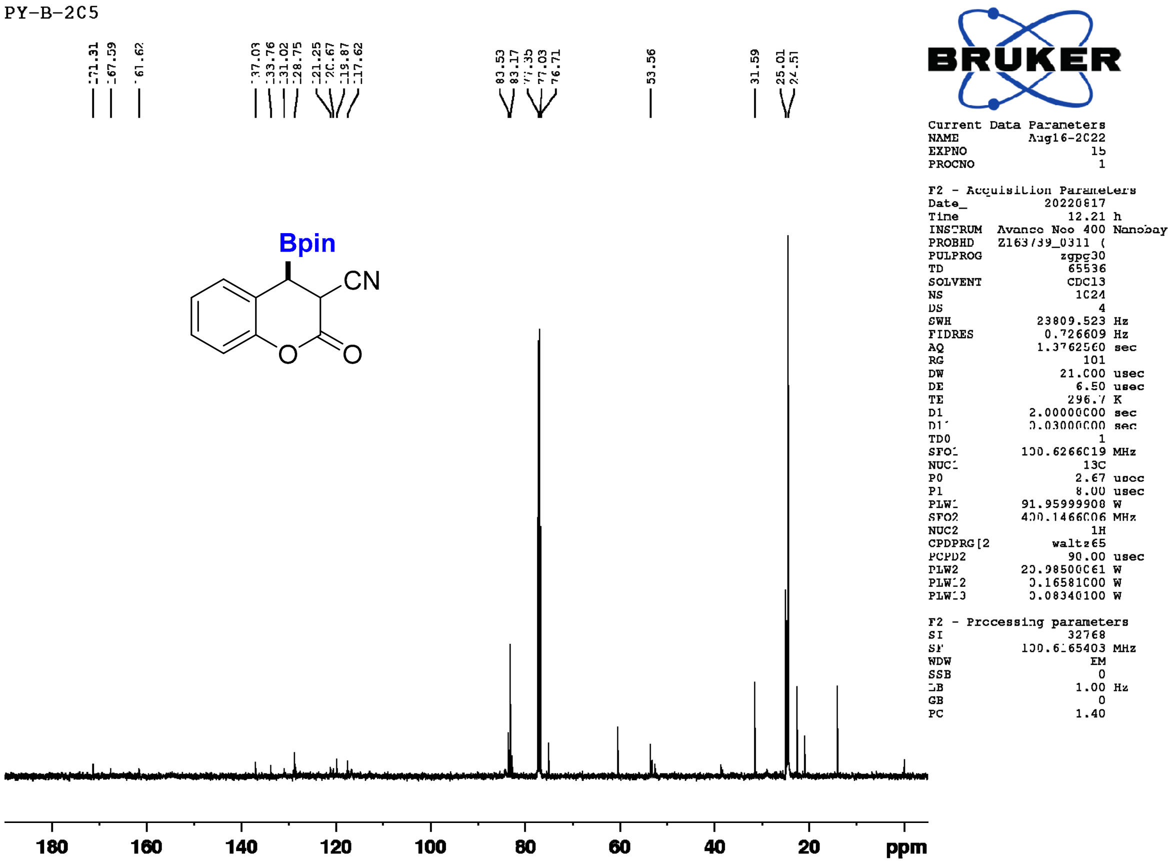

^{13}C NMR spectrum of **2d**

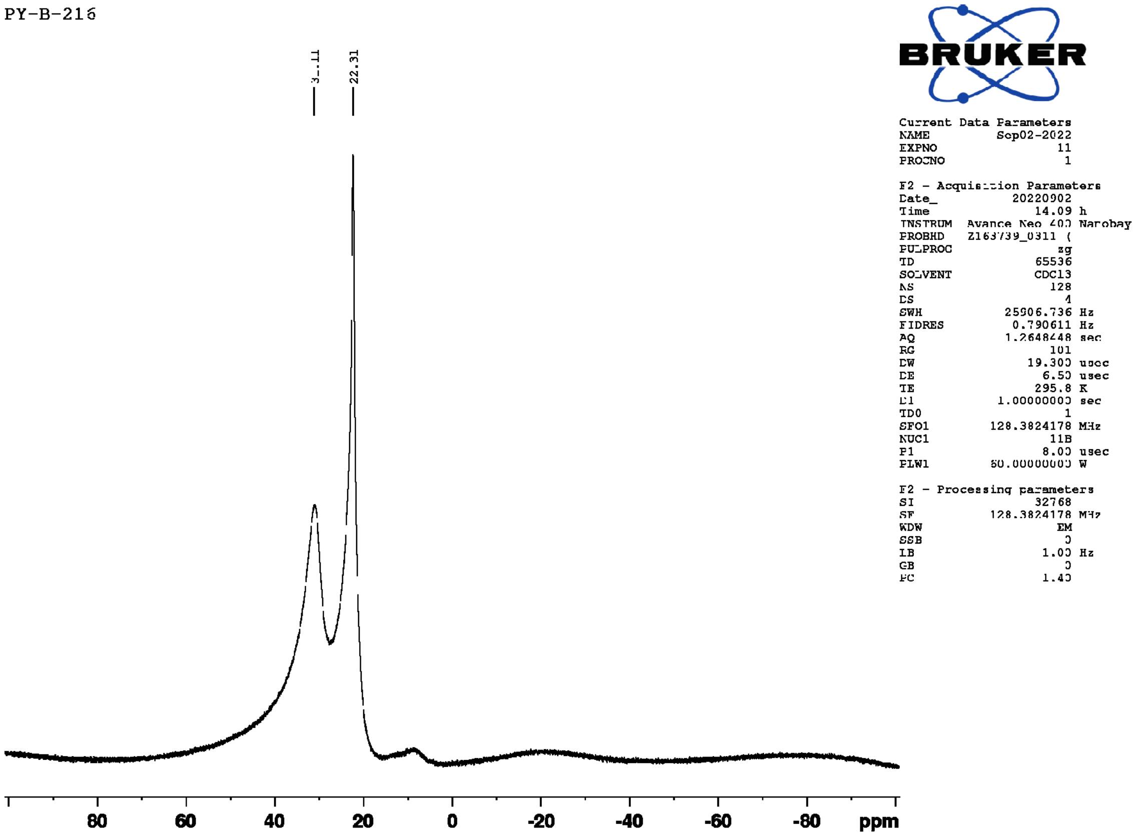

^{11}B NMR spectrum of **2d**

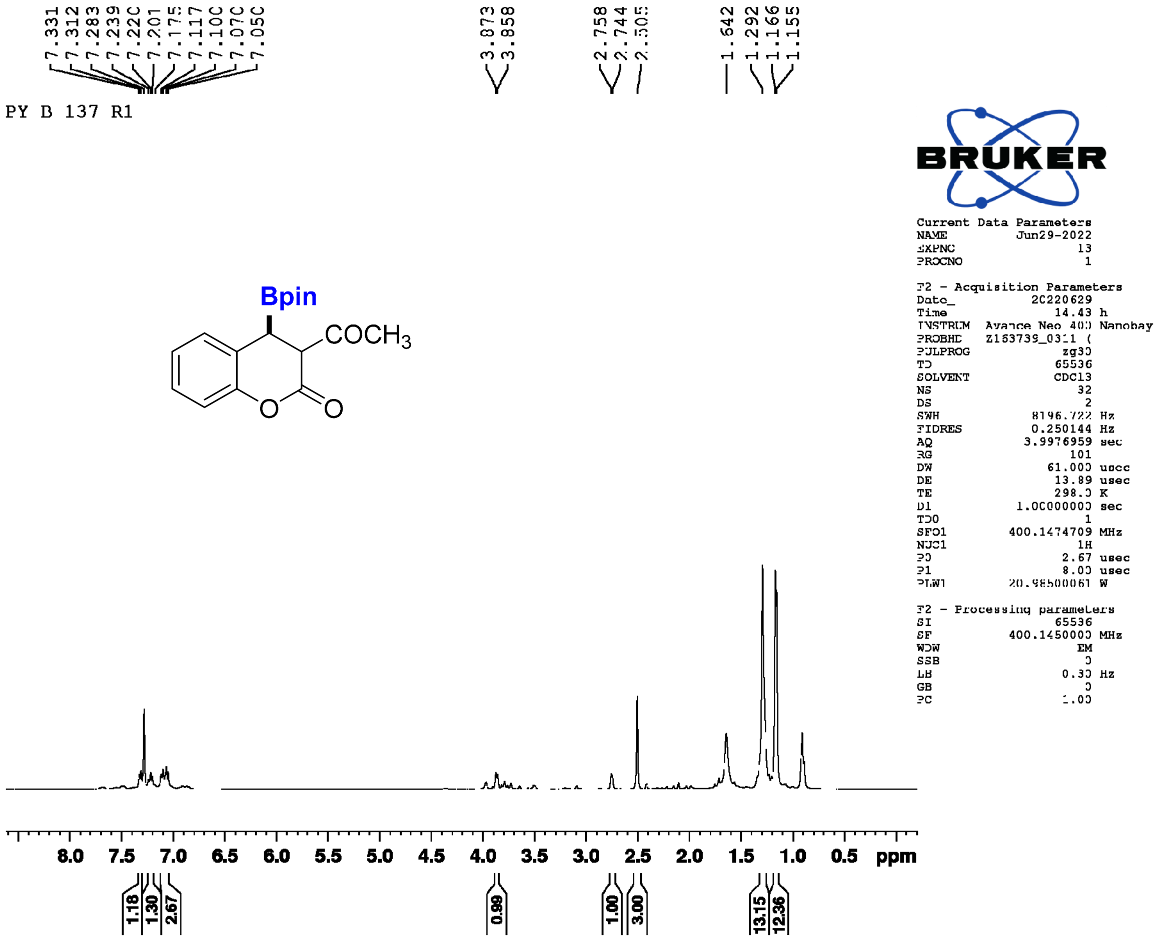

^{1}H NMR spectrum of **2e**

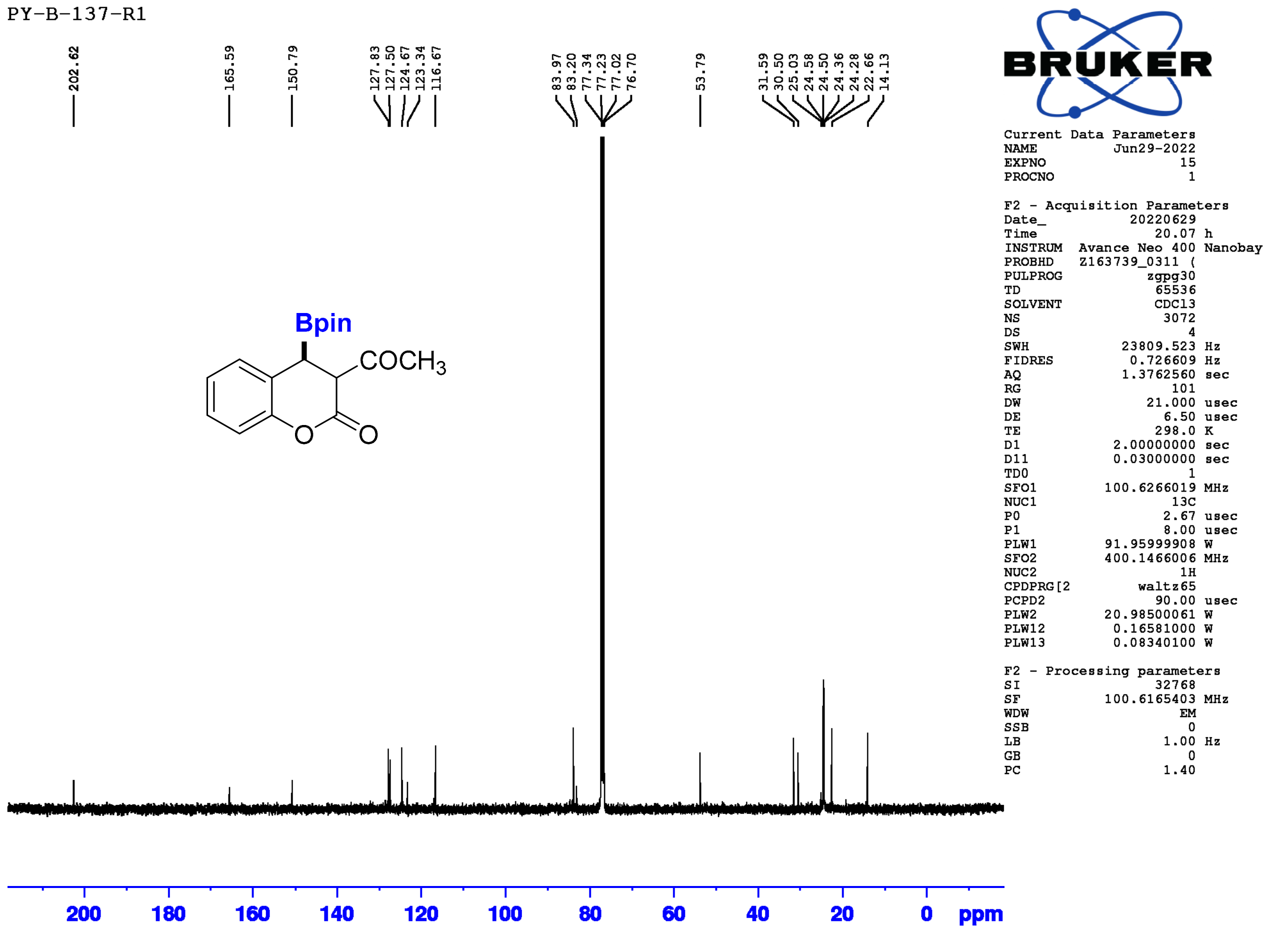

^{13}C NMR spectrum of **2e**

Spectrum View - PY-B-211ketone.d

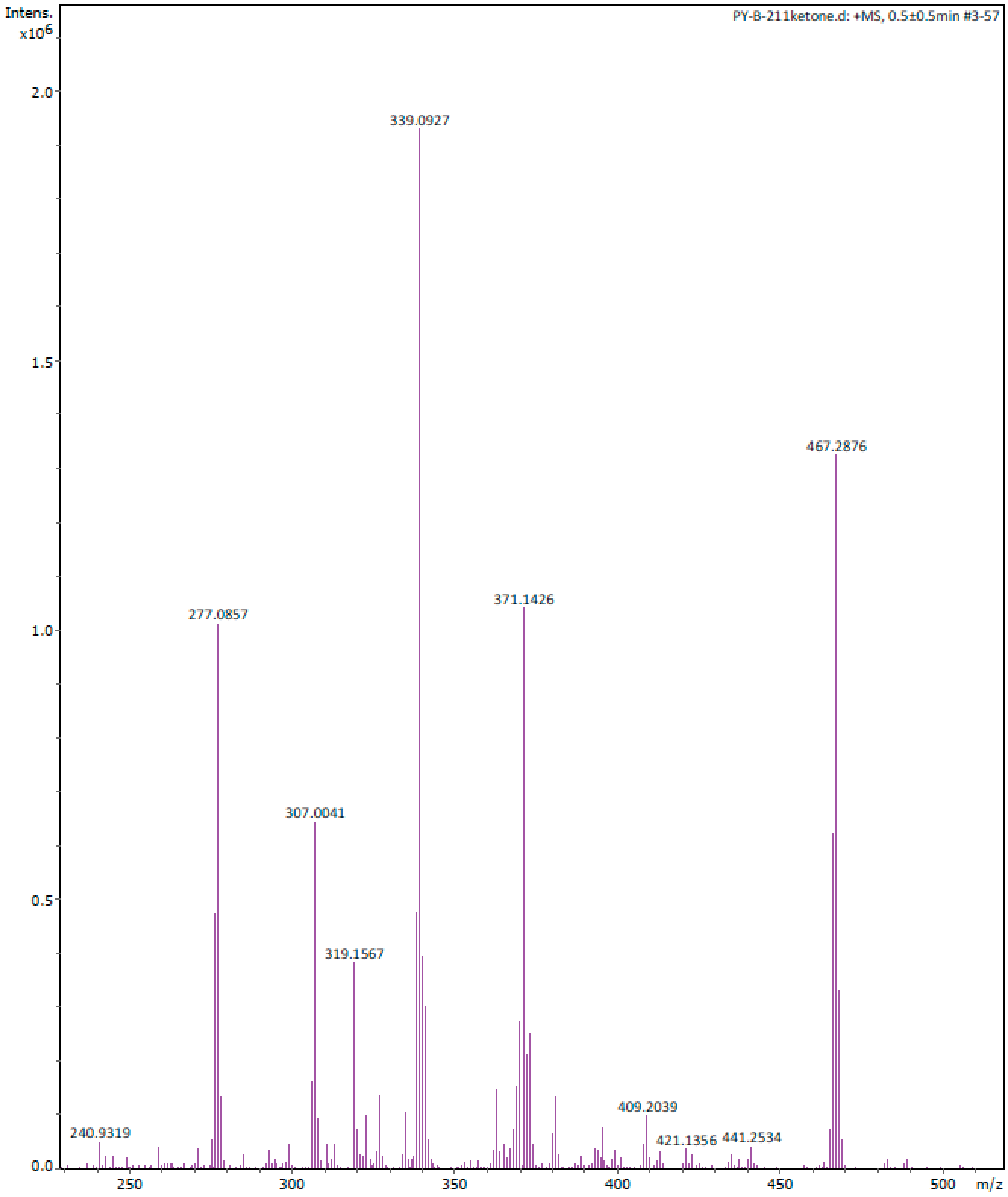

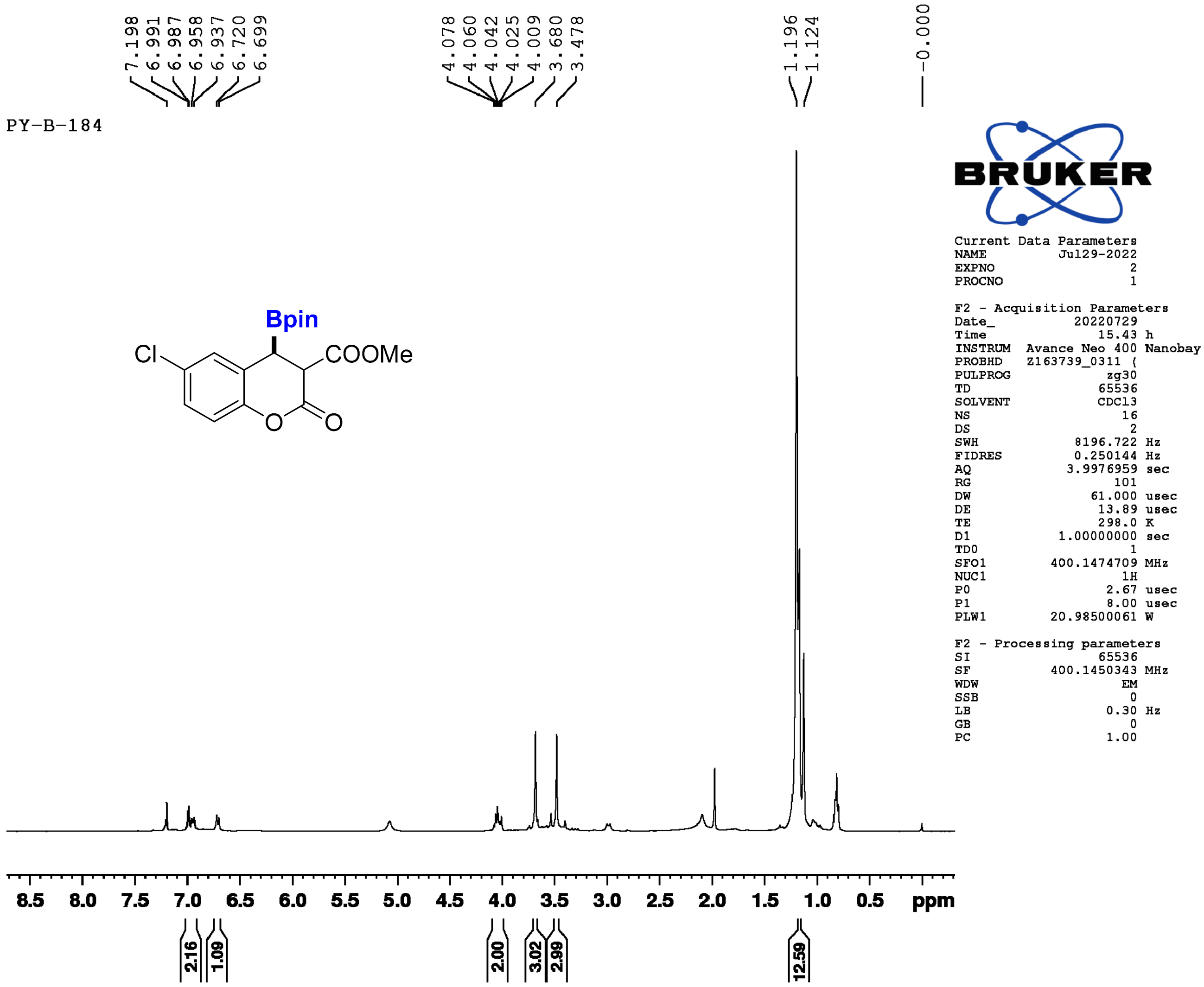

^{1}H NMR spectrum of **2f**

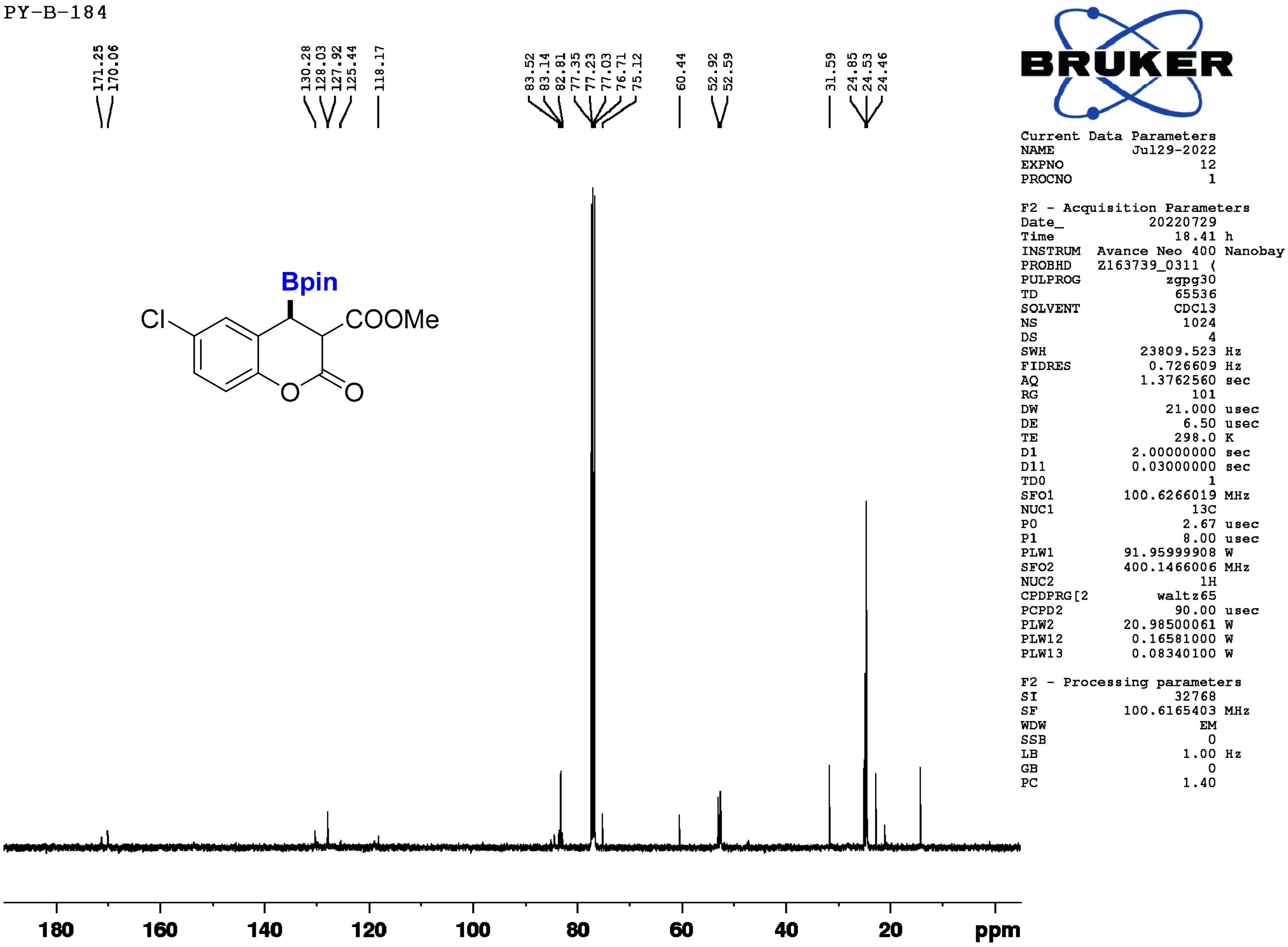

^{13}C NMR spectrum of **2f**

Spectrum View - PY-B-212 cl ester.d

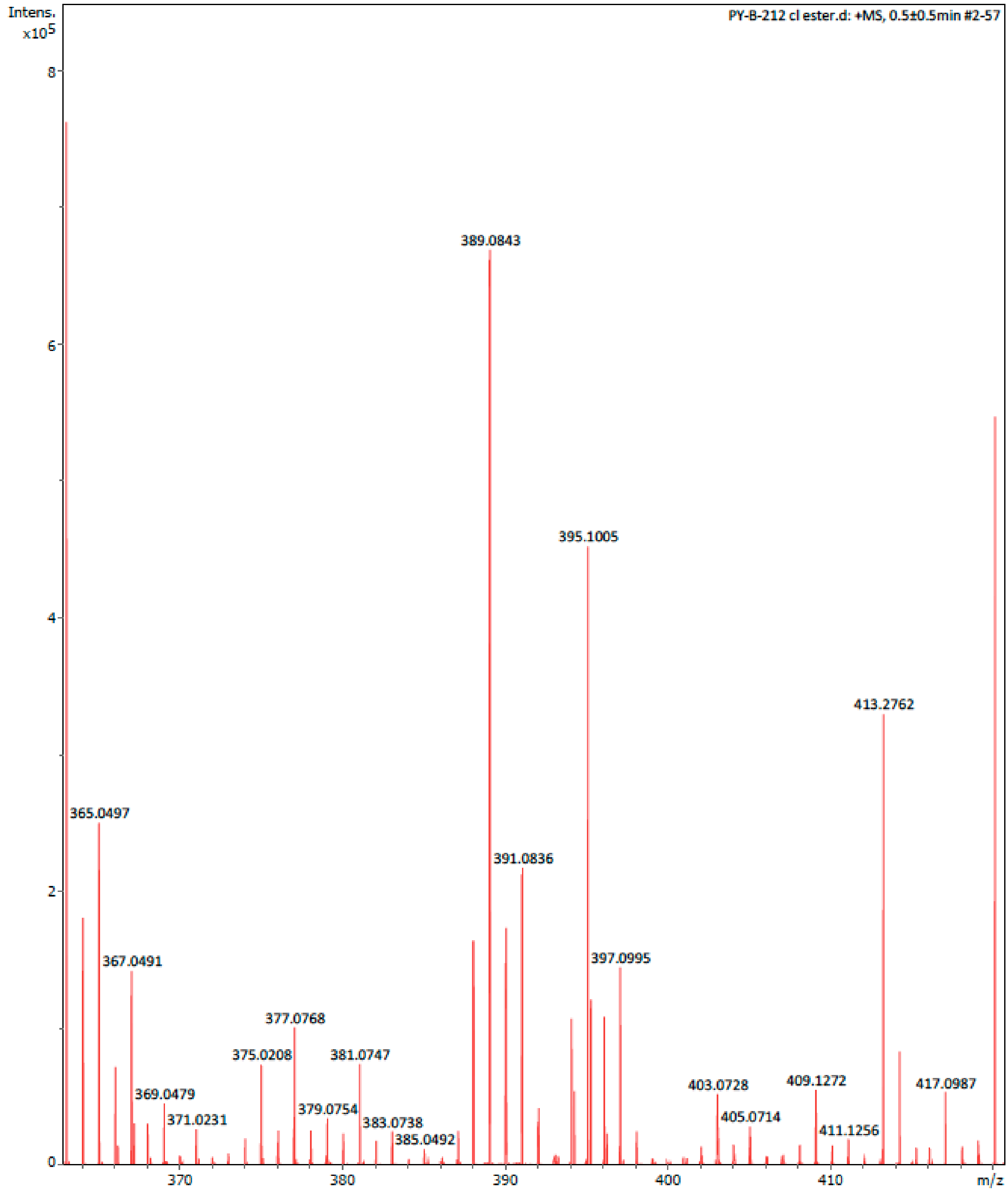

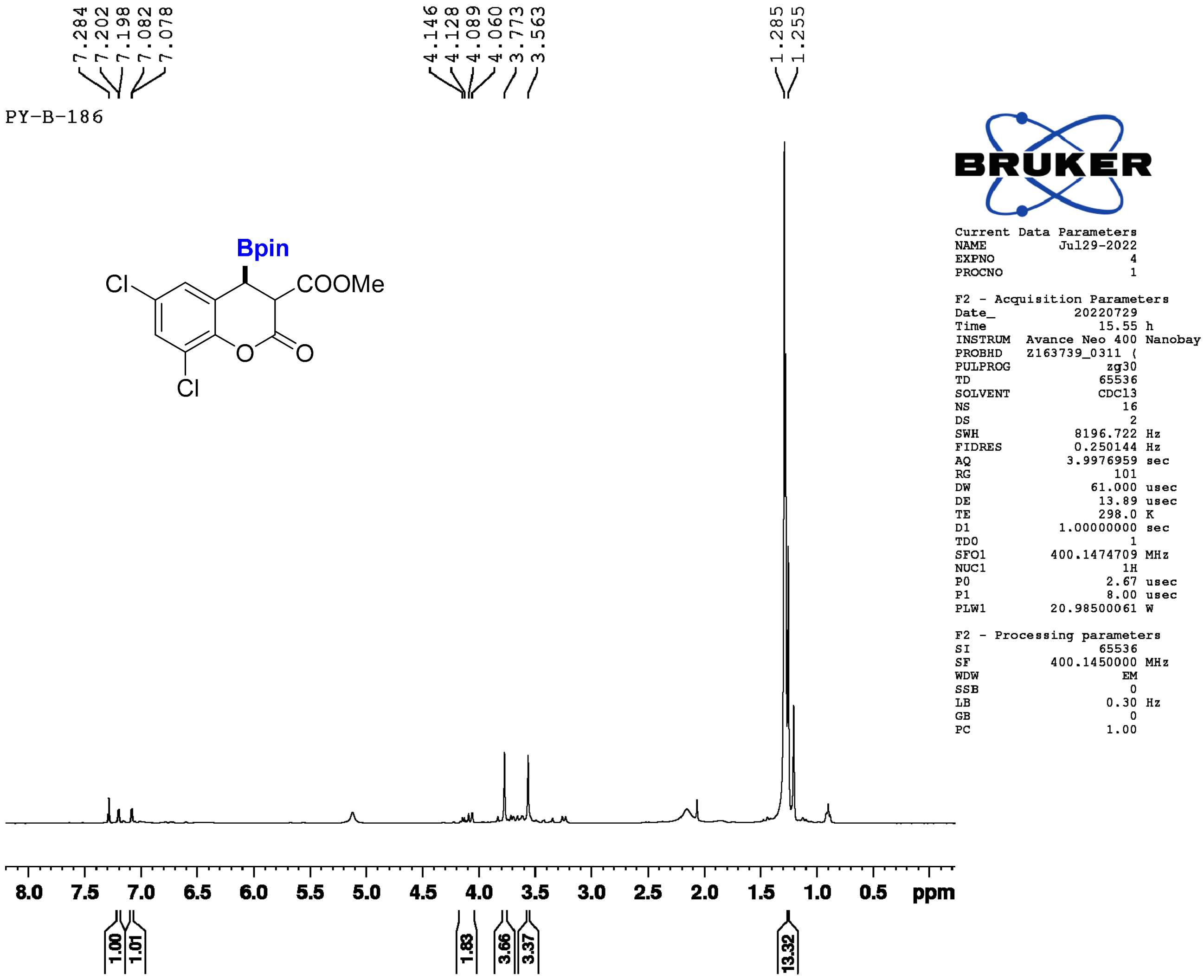

^{1}H NMR spectrum of **2g**

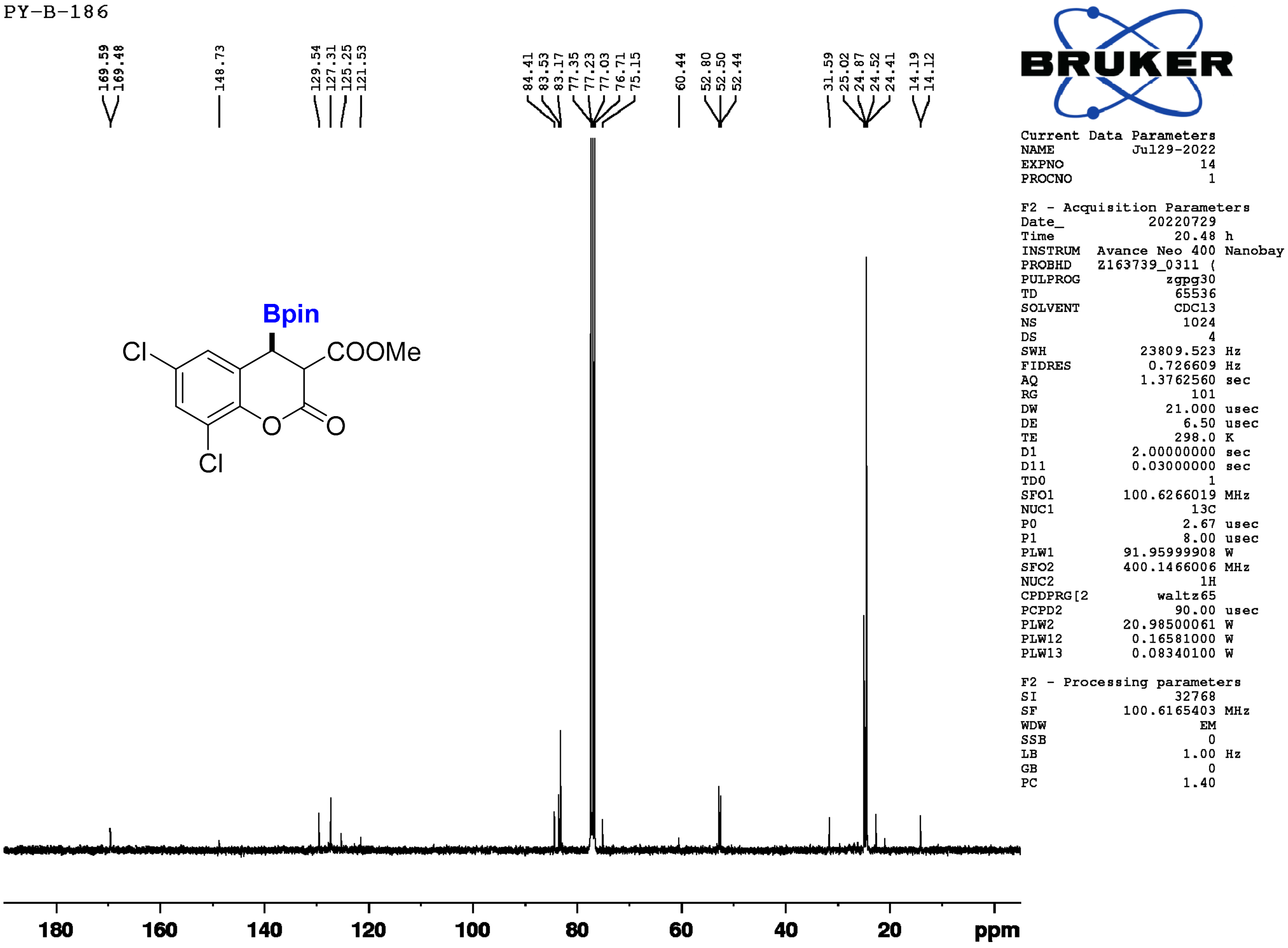

^{13}C NMR spectrum of **2g**

Window Display Report

Spectrum View - PY-B-217 METHYL ESTER 2-CL.d

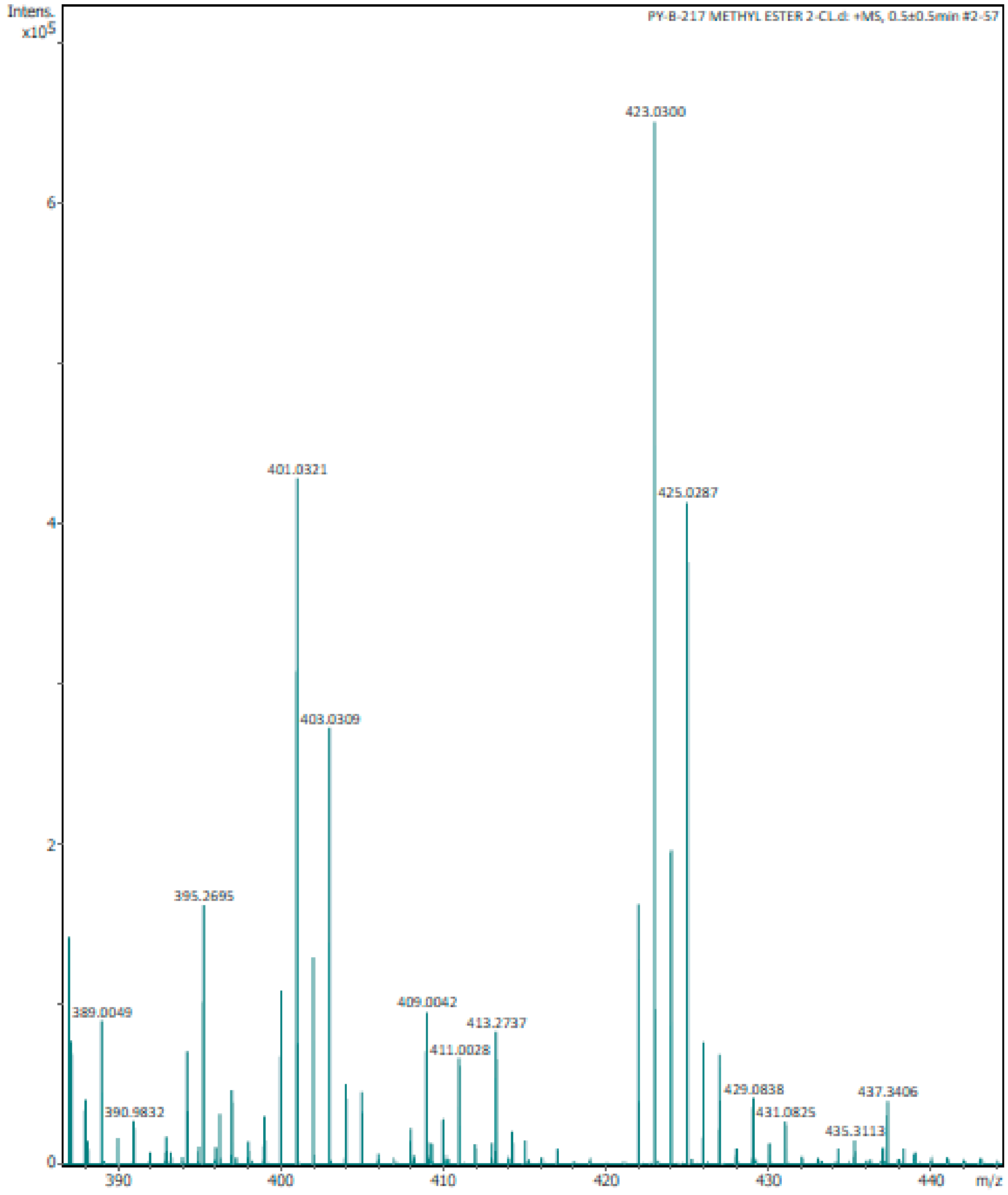

Bruker Compass DataAnalysis 5.2 printed: 9/15/2022 1:11:50 PM by: demo Page 1 of 1

Mass spectrum of **2g**

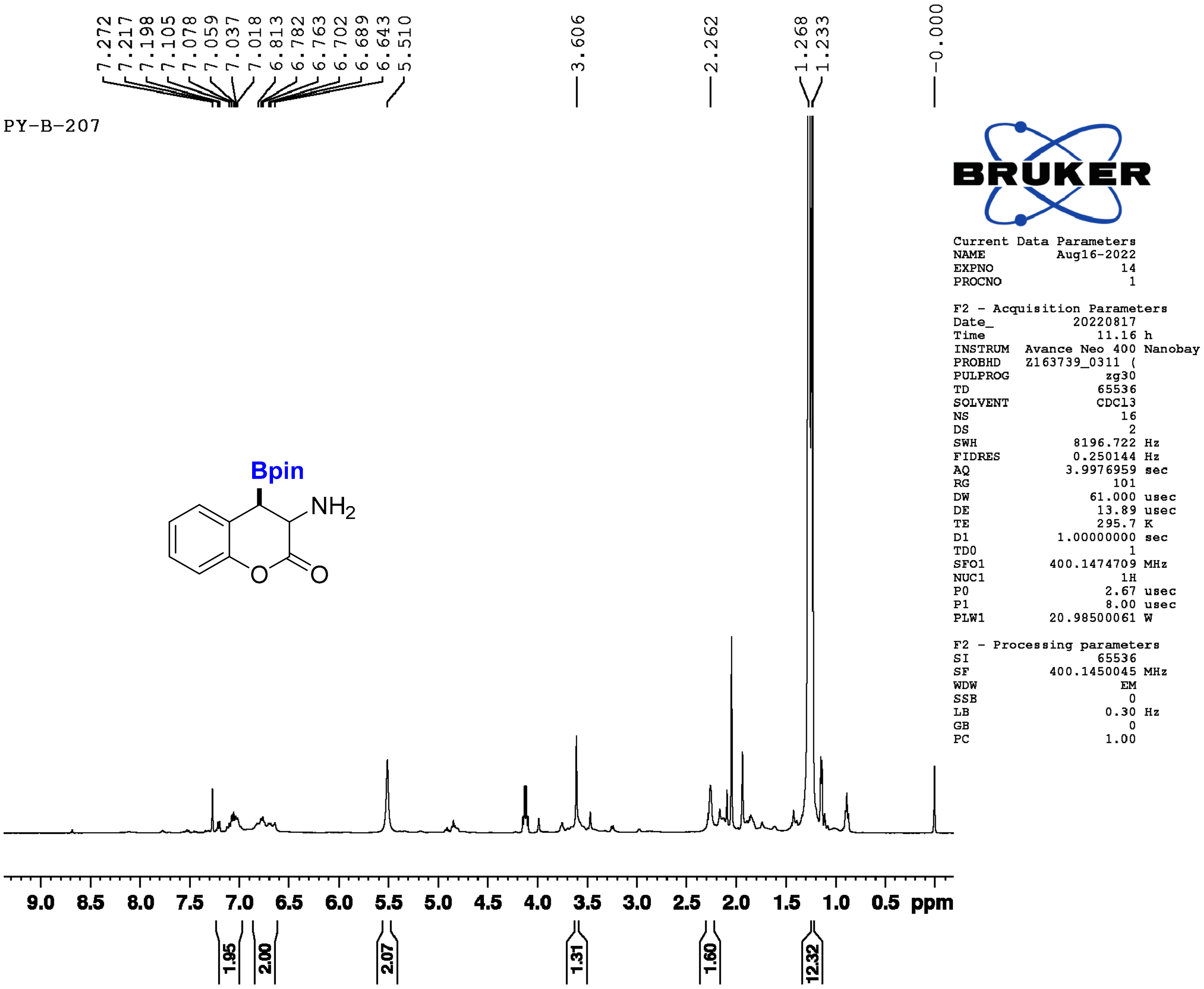

^{1}H NMR spectrum of **2h**

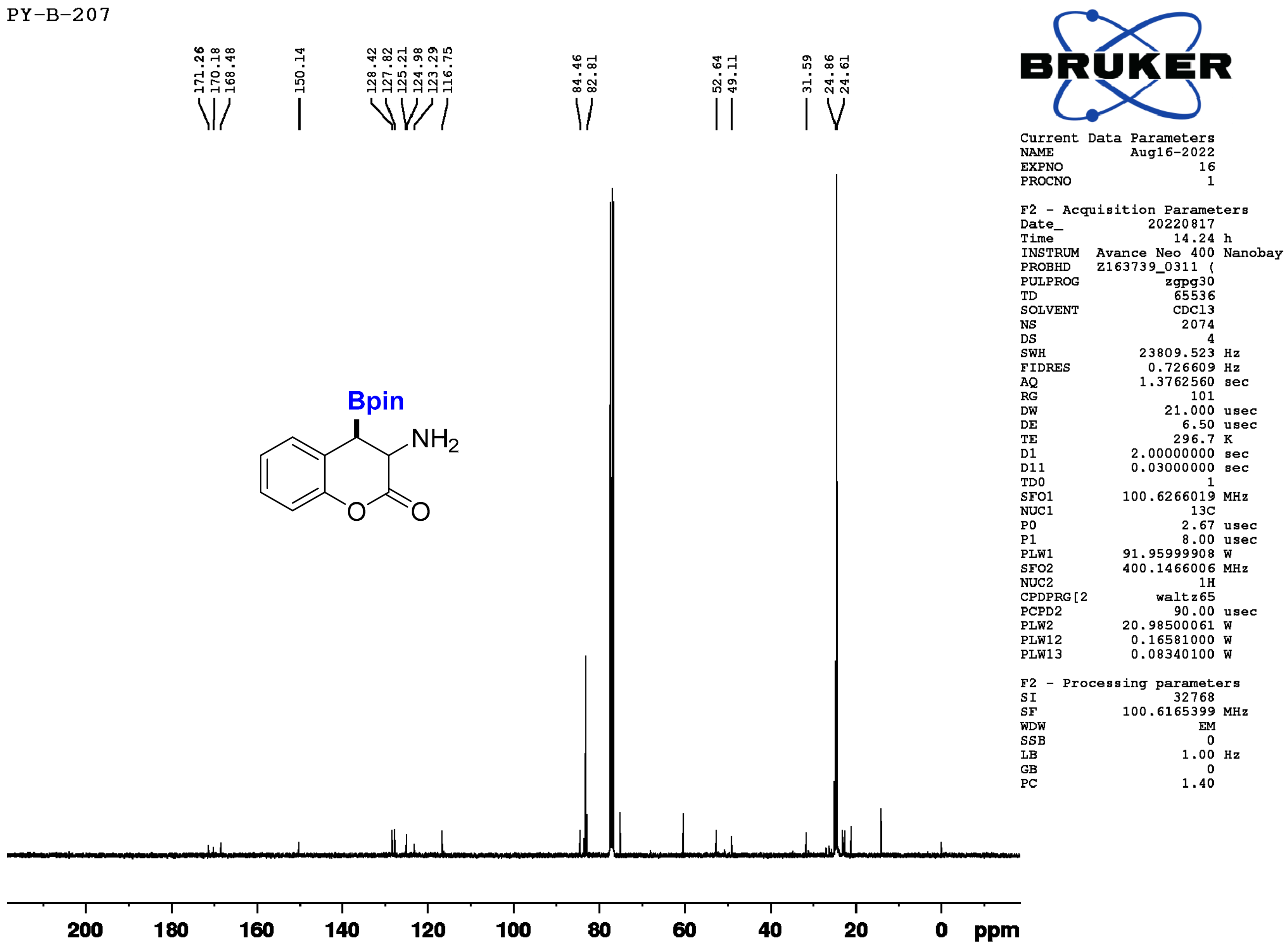

^{13}C NMR spectrum of **2h**

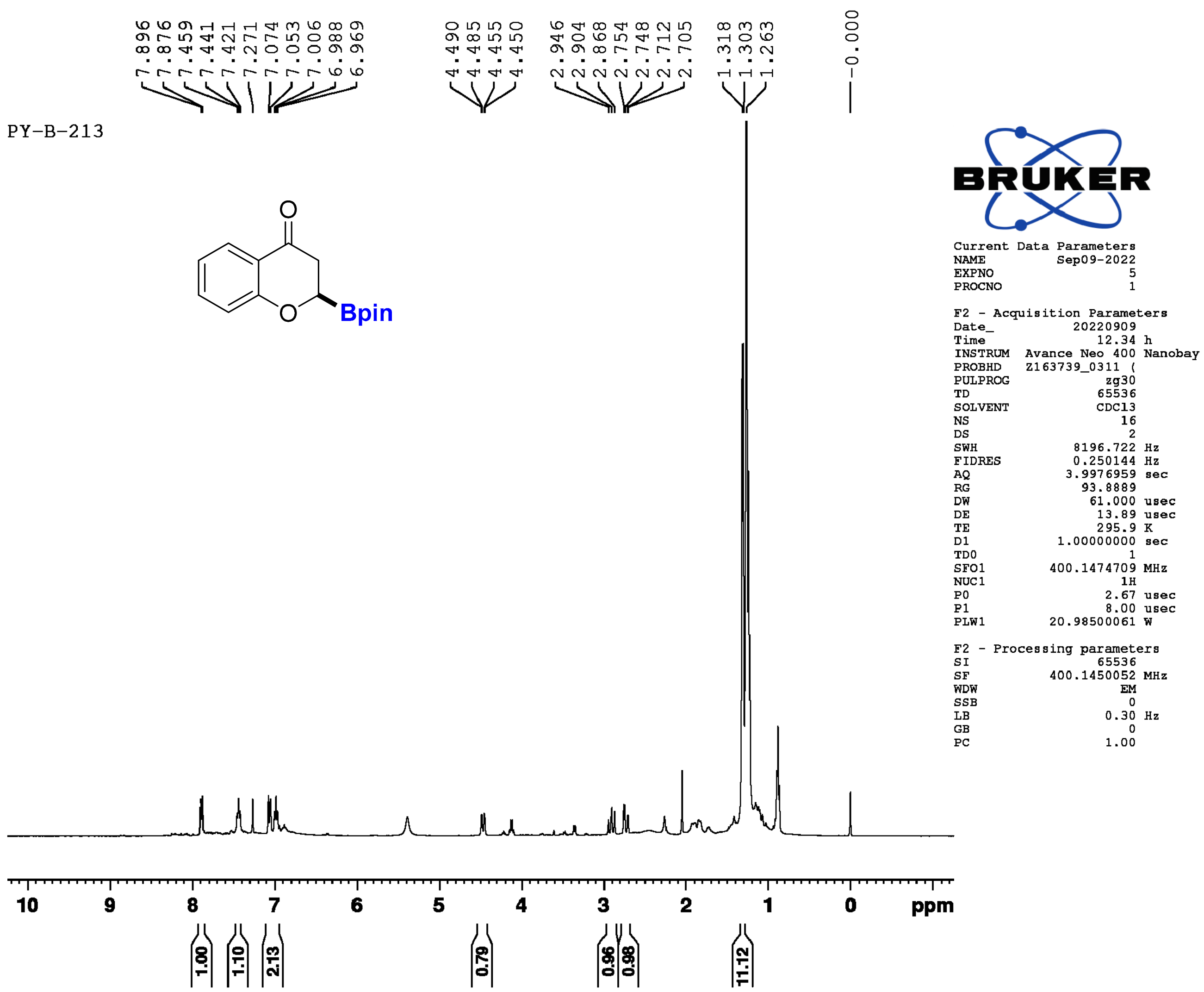

^{1}H NMR spectrum of **4a**

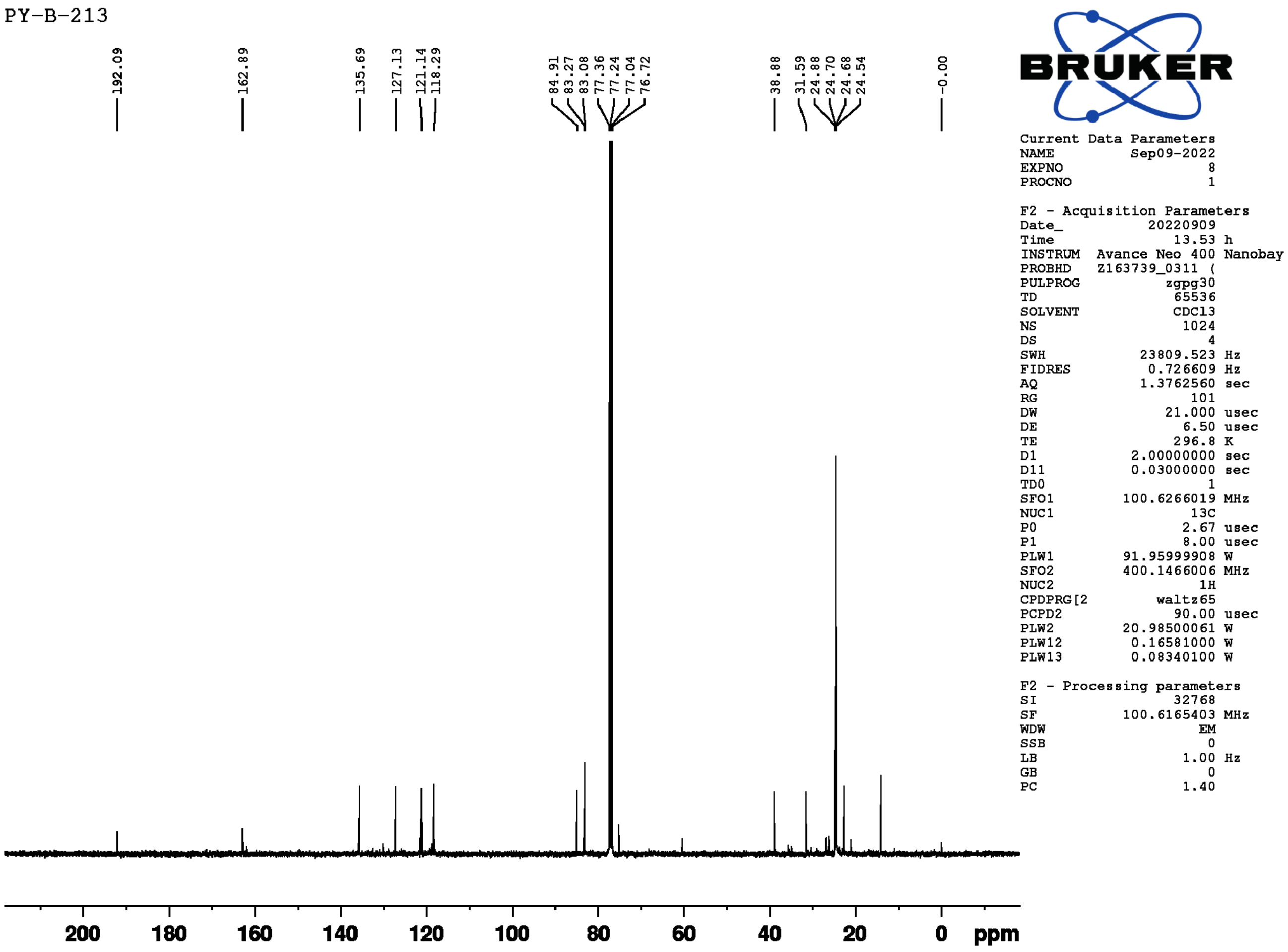

^{13}C NMR spectrum of **4a**

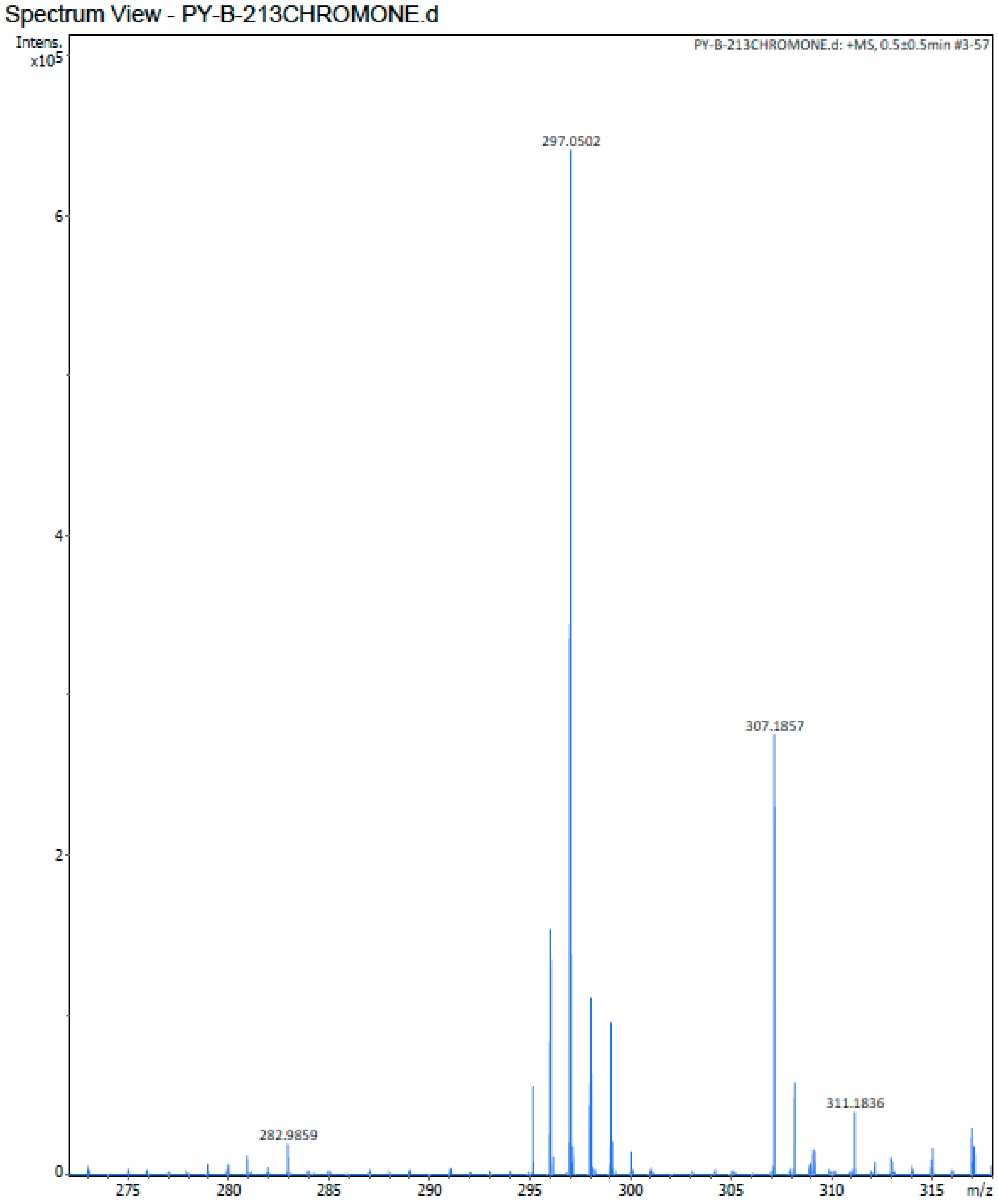

Mass spectrum of **4a**

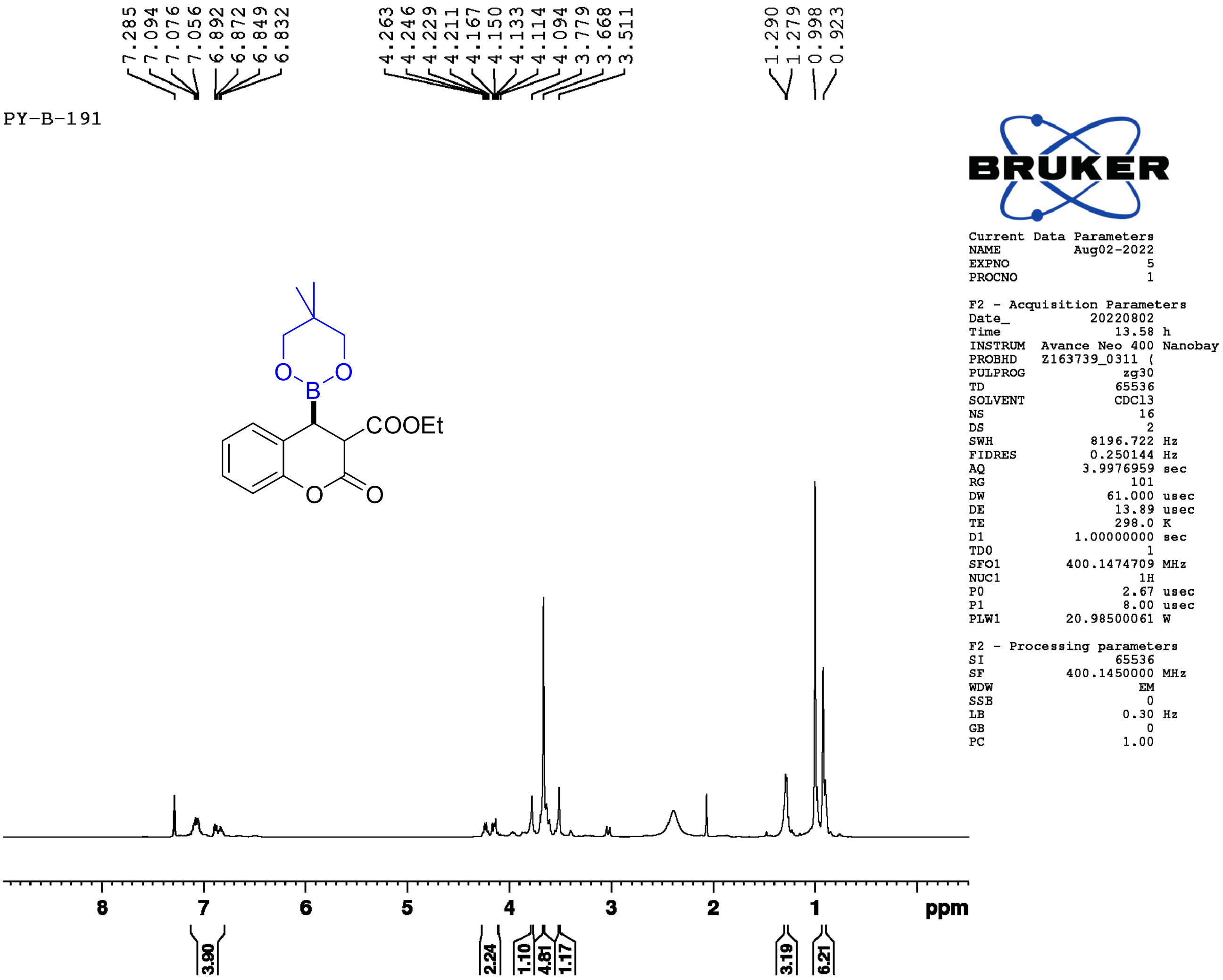

^{1}H NMR spectrum of **4b**

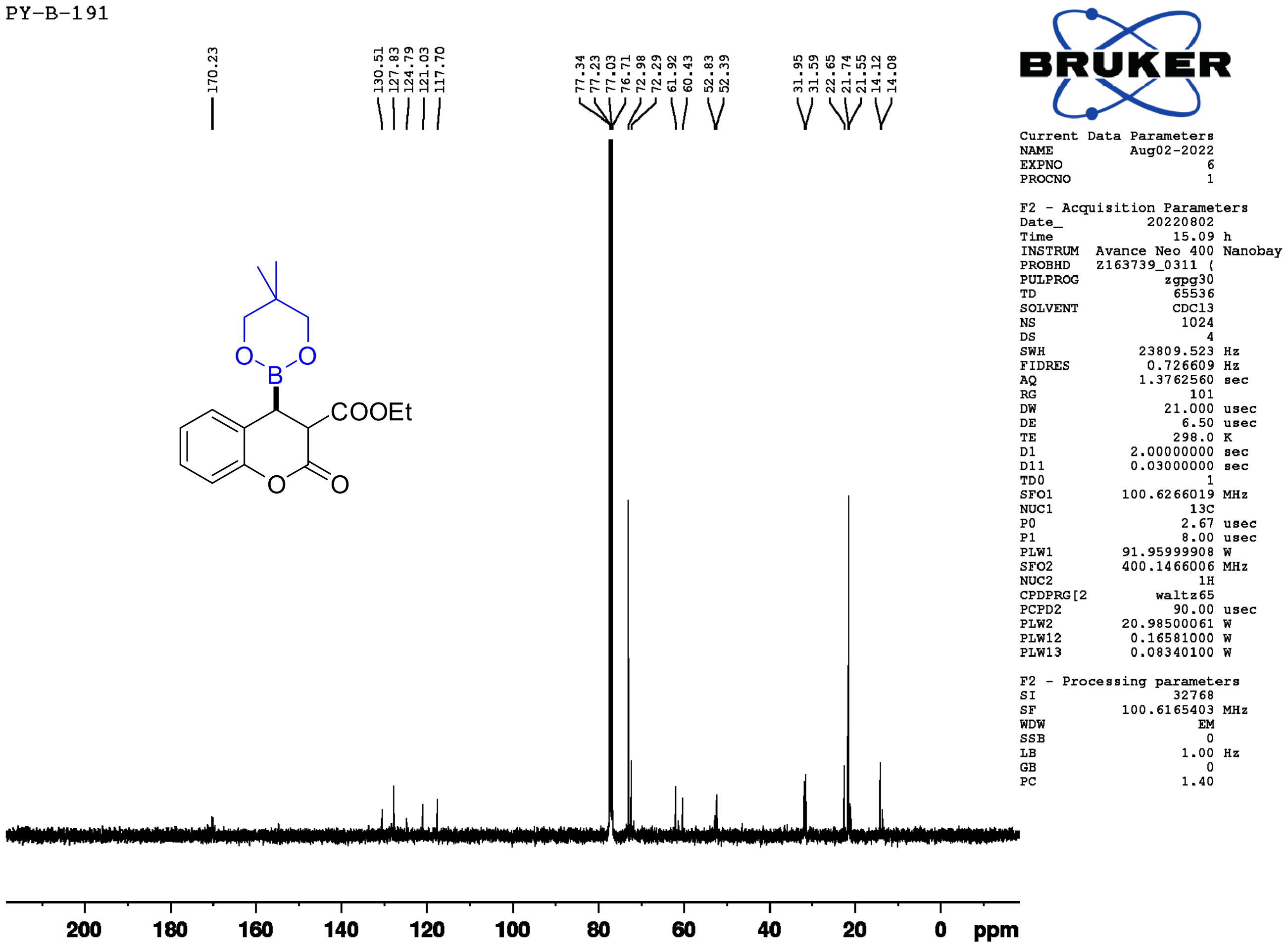

^{13}C NMR spectrum of **4b**

Window Display Report

Spectrum View - PY-B-221R.d

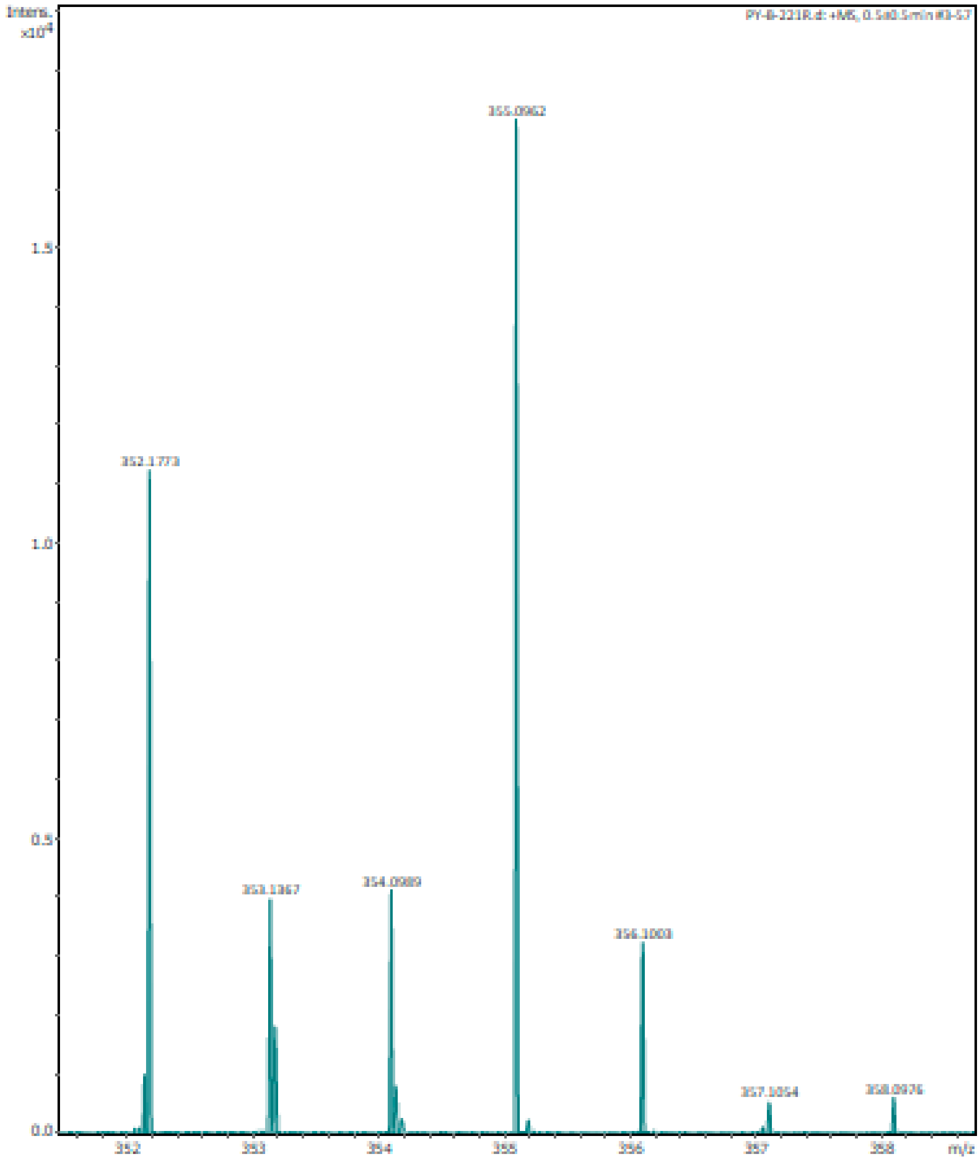

Bruker Compass DataAnalysis 5.2 printed: 9/16/2022 1:02:53 PM by: demo Page 1 of 1

Mass spectrum of **4b**

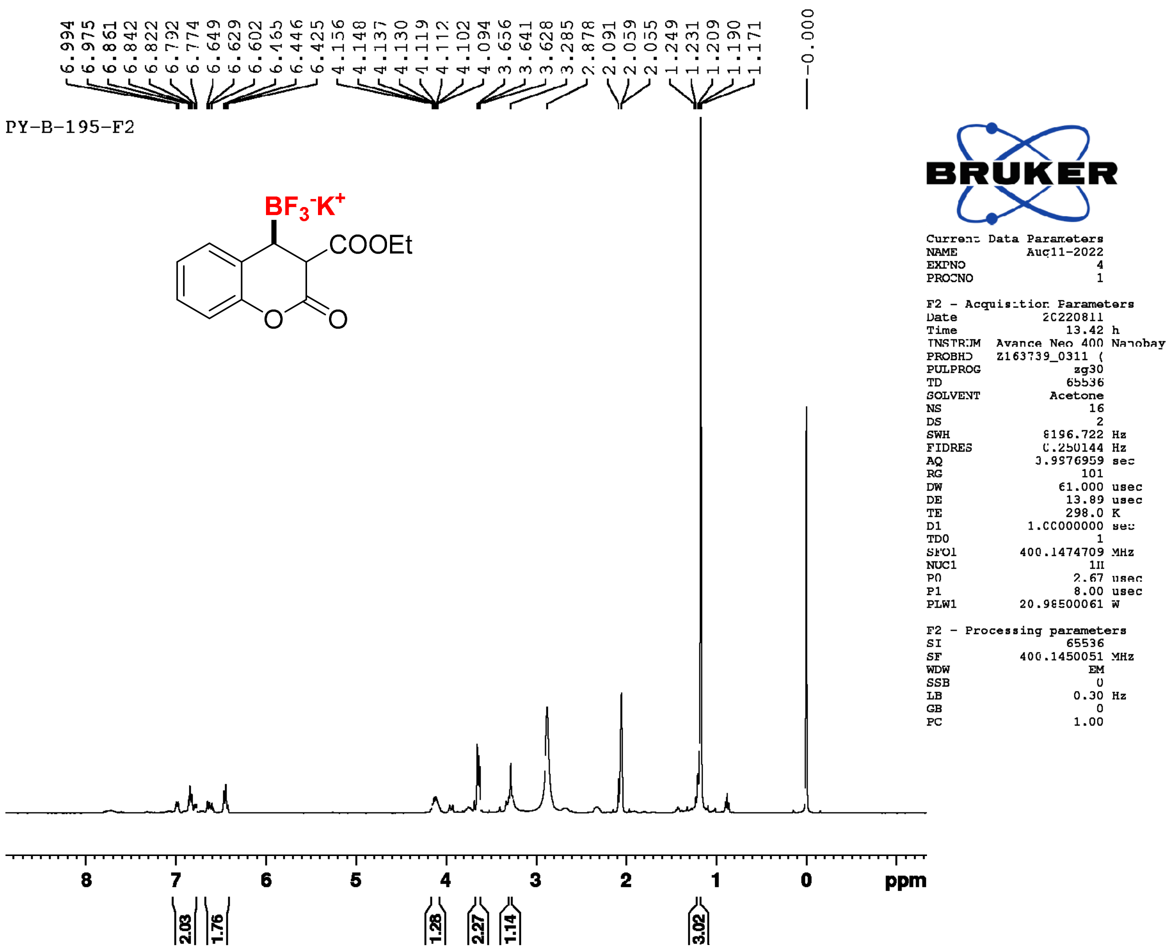

^{1}H NMR spectrum of **5a**

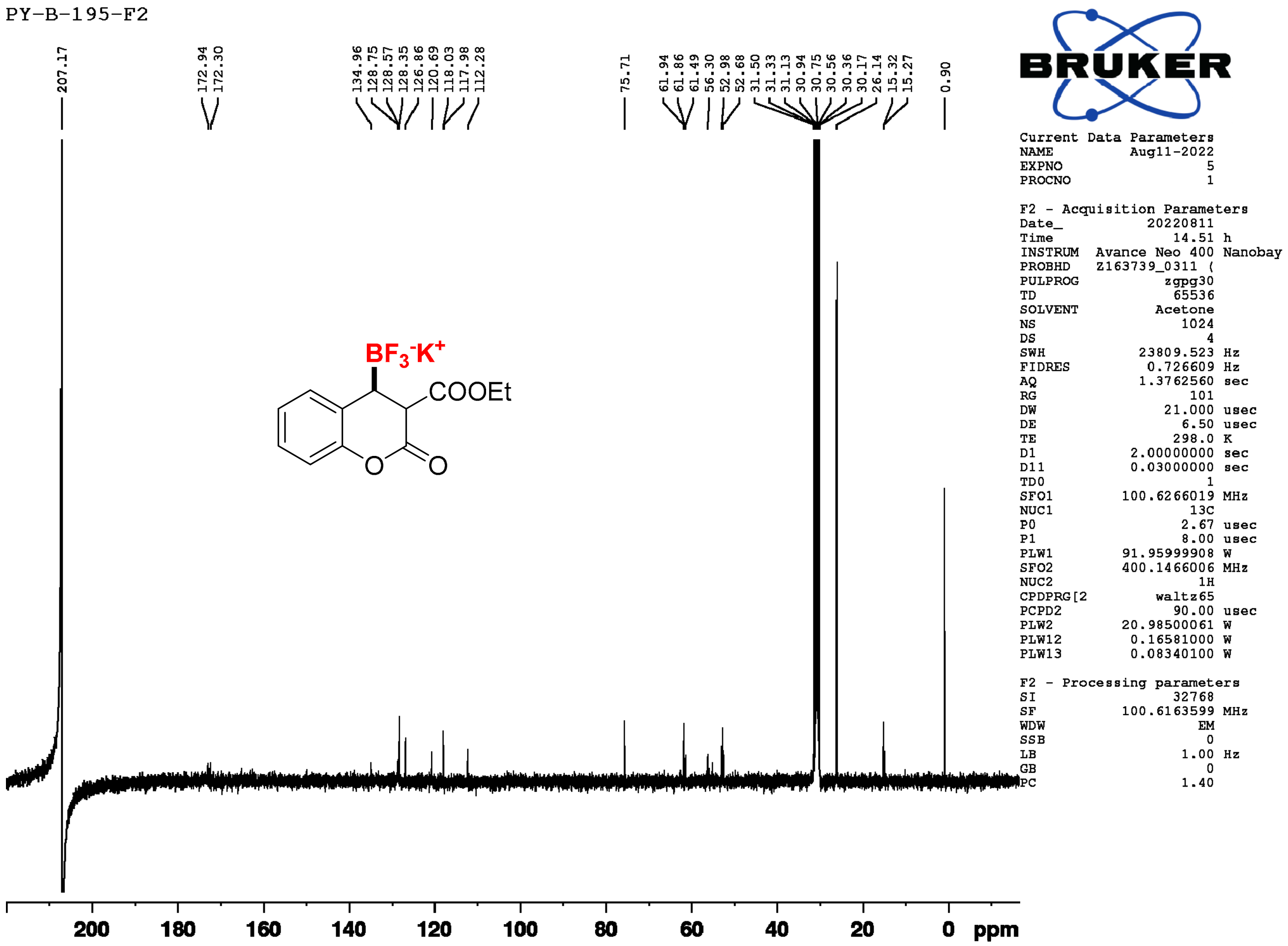

^{13}C NMR spectrum of **5a**

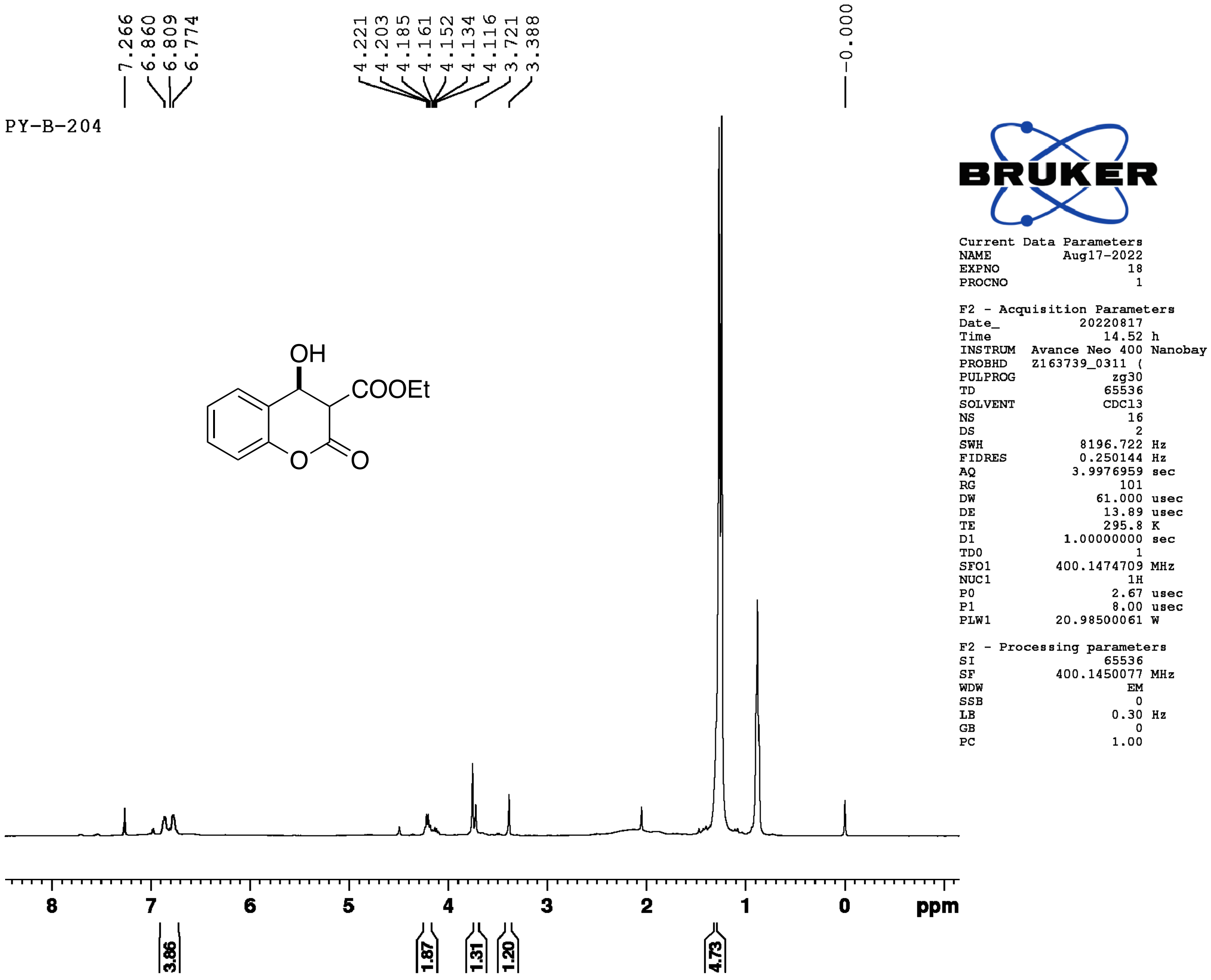

^{1}H NMR spectrum of **5b**

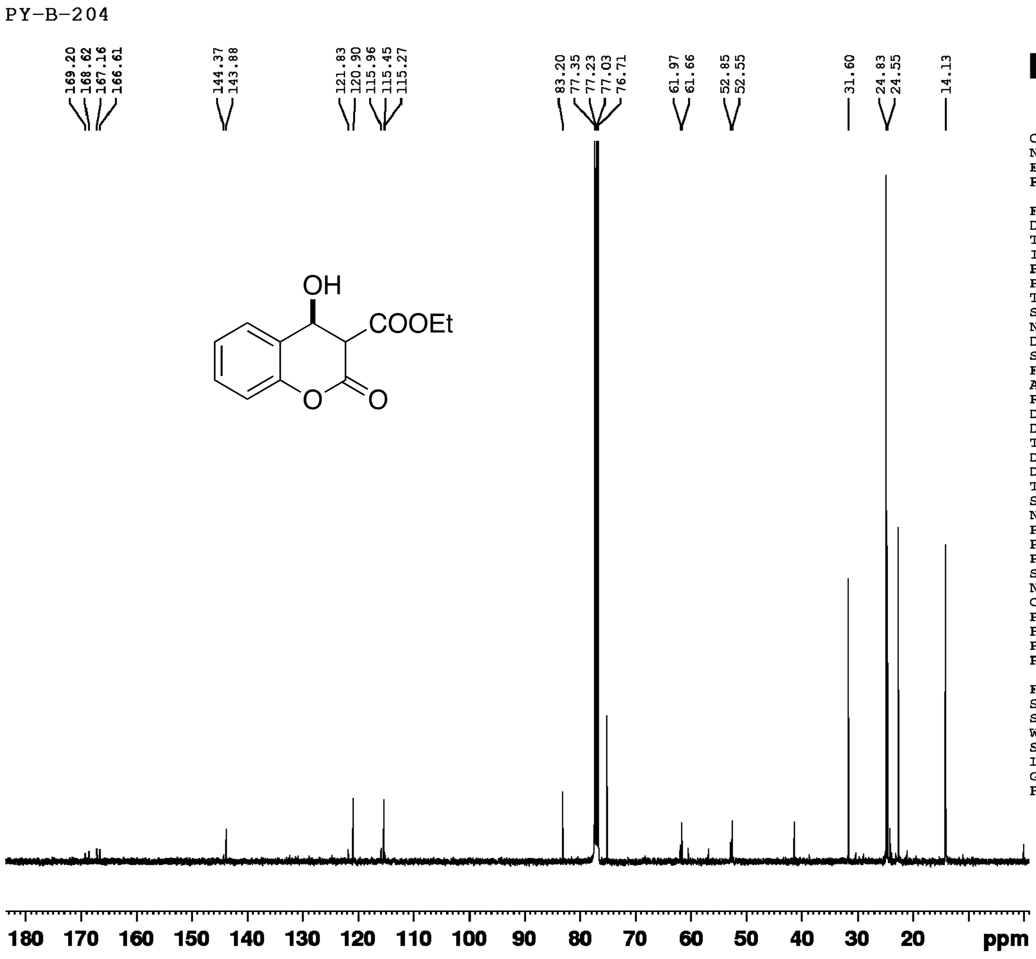


```
Current Data Parameters
NAME          Aug17-2022
EXPNO                 20
PROCNO                 1

F2 - Acquisition Parameters
Date_           20220817
Time               17.59 h
INSTRUM   Avance Neo 400 Nanobay
PROBHD    Z163739_0311 (
PULPROG           zgpg30
TD                 65536
SOLVENT            CDCl3
NS                  3072
DS                     4
SWH            23809.523 Hz
FIDRES          0.726609 Hz
AQ             1.3762560 sec
RG                   101
DW                21.000 usec
DE                  6.50 usec
TE                 296.7 K
D1            2.00000000 sec
D11           0.03000000 sec
TD0                    1
SFO1         100.6266019 MHz
NUC1                 13C
P0                  2.67 usec
P1                  8.00 usec
PLW1         91.95999908 W
SFO2         400.1466006 MHz
NUC2                  1H
CPDPRG[2         waltz65
PCPD2              90.00 usec
PLW2         20.98500061 W
PLW12         0.16581000 W
PLW13         0.08340100 W

F2 - Processing parameters
SI                 32768
SF           100.6165403 MHz
WDW                   EM
SSB                    0
LB                  1.00 Hz
GB                     0
PC                  1.40
```

^{13}C NMR spectrum of **5b**

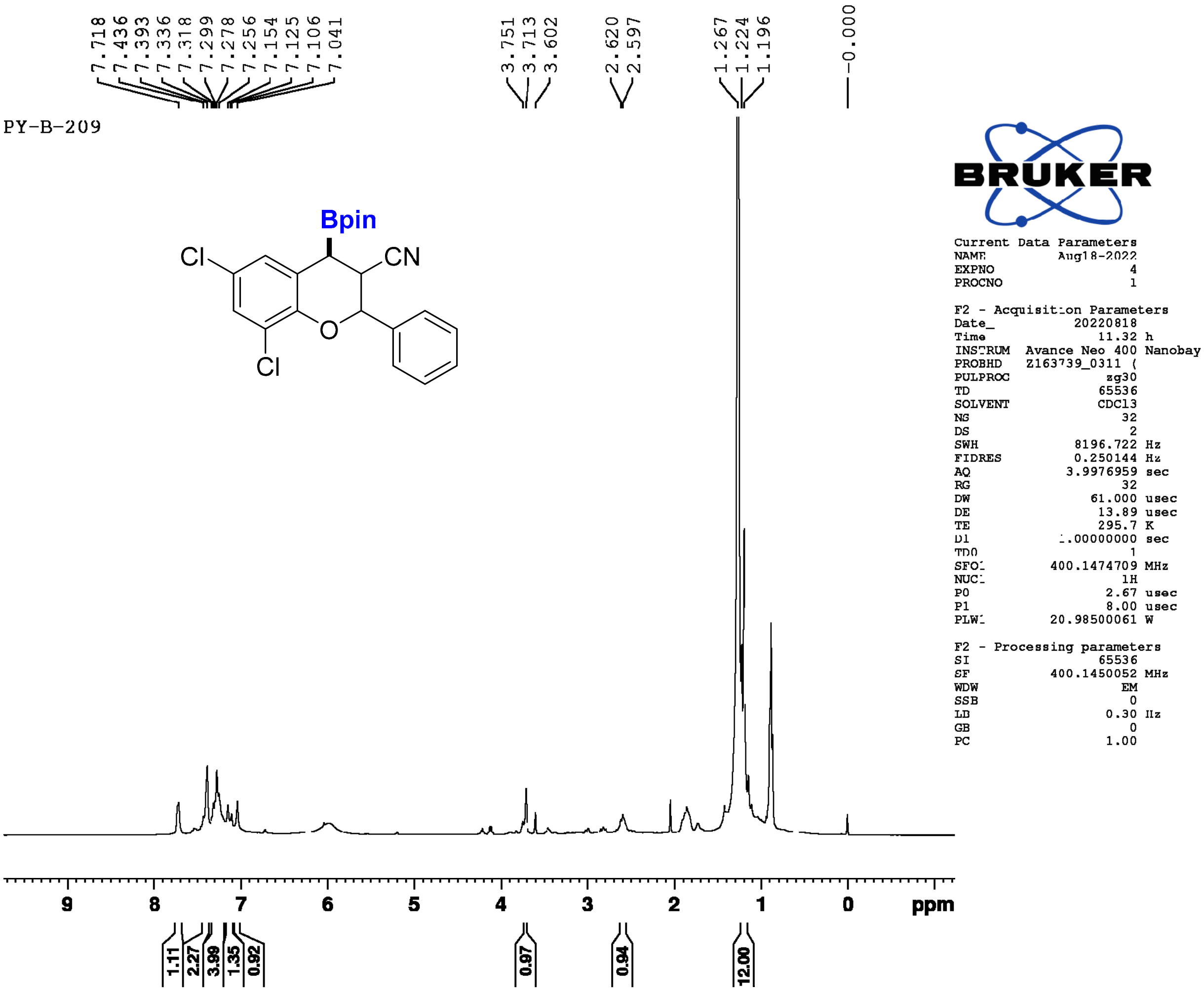

^{1}H NMR spectrum of **7a**

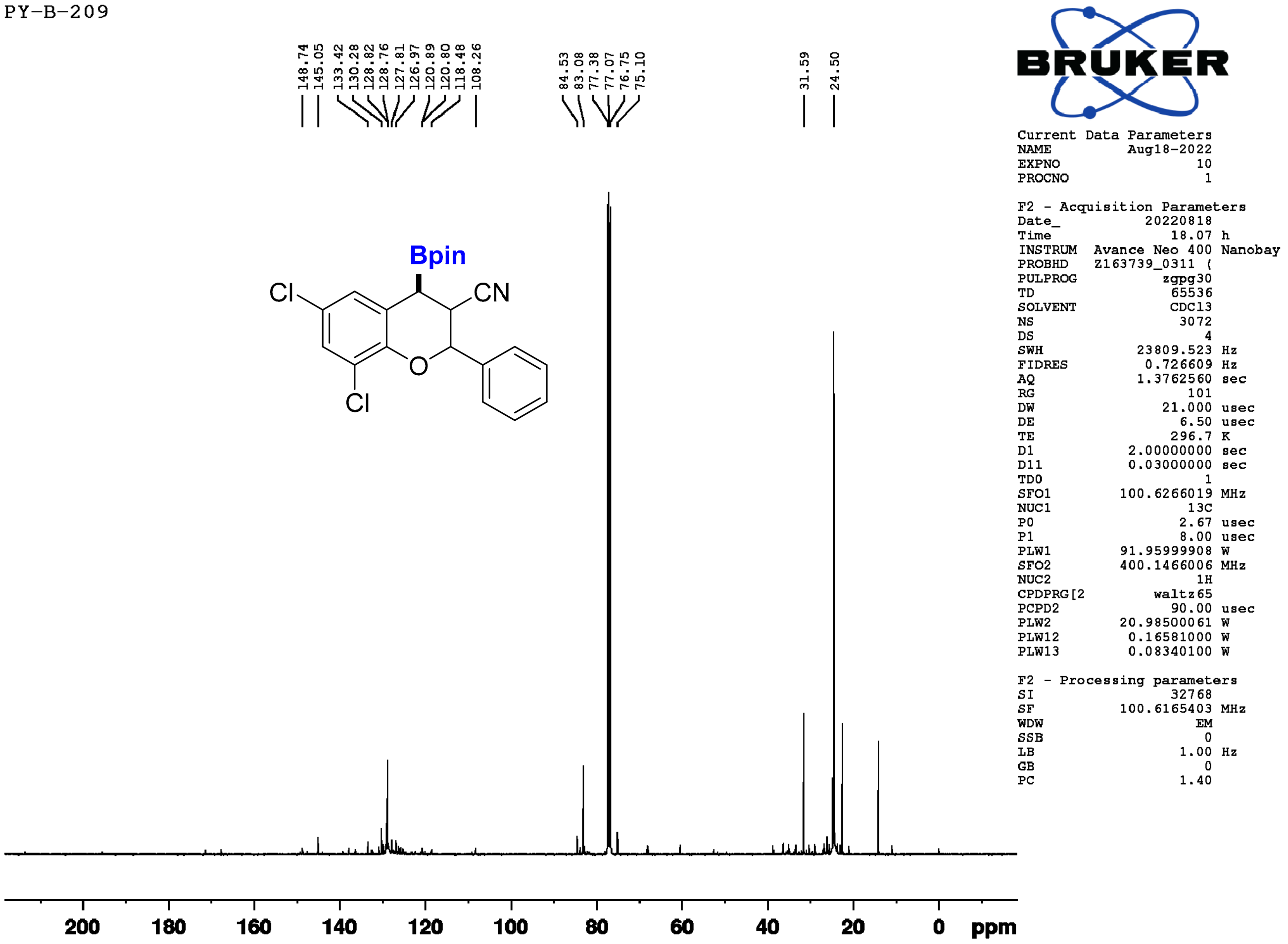

^{13}C NMR spectrum of **7a**

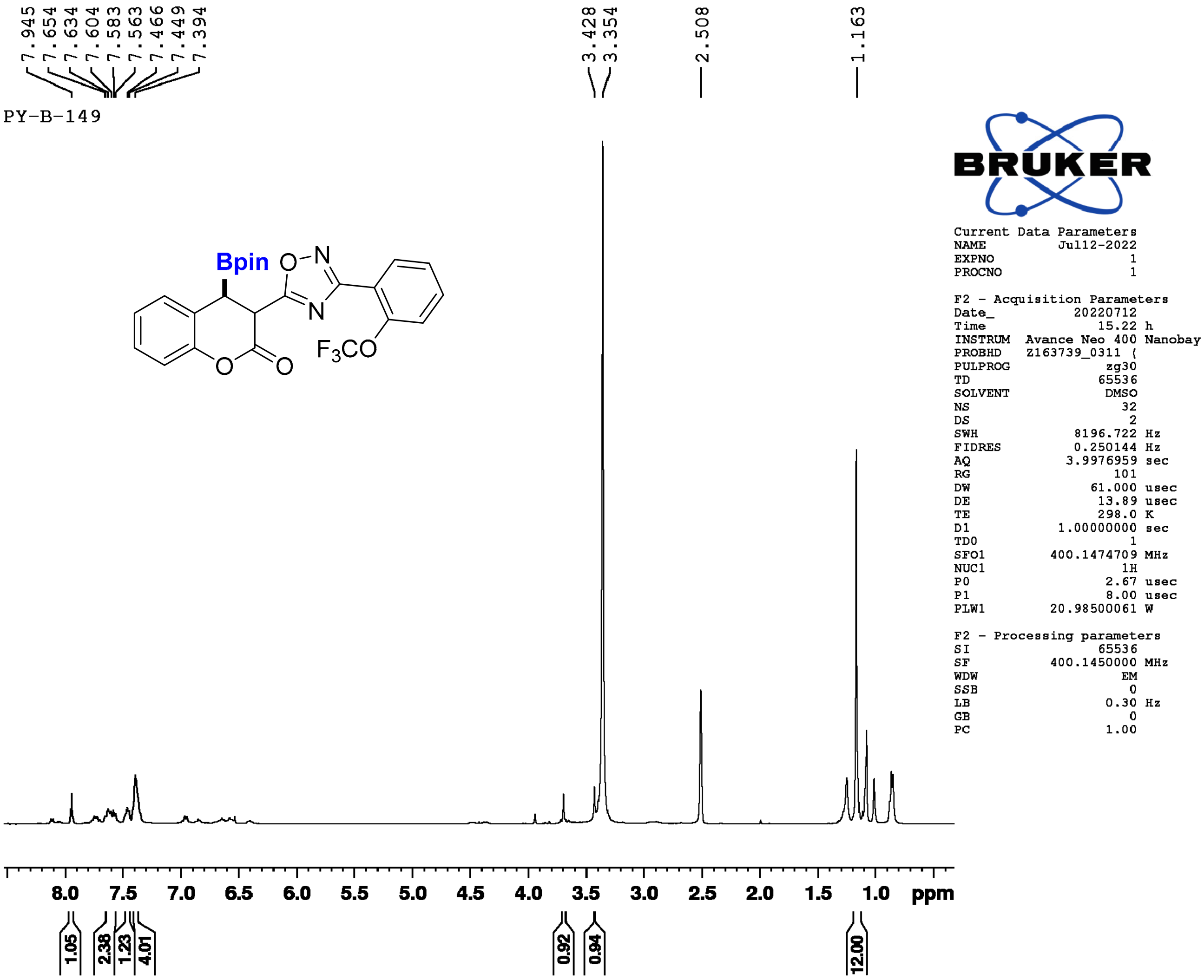

^{1}H NMR spectrum of **7b**

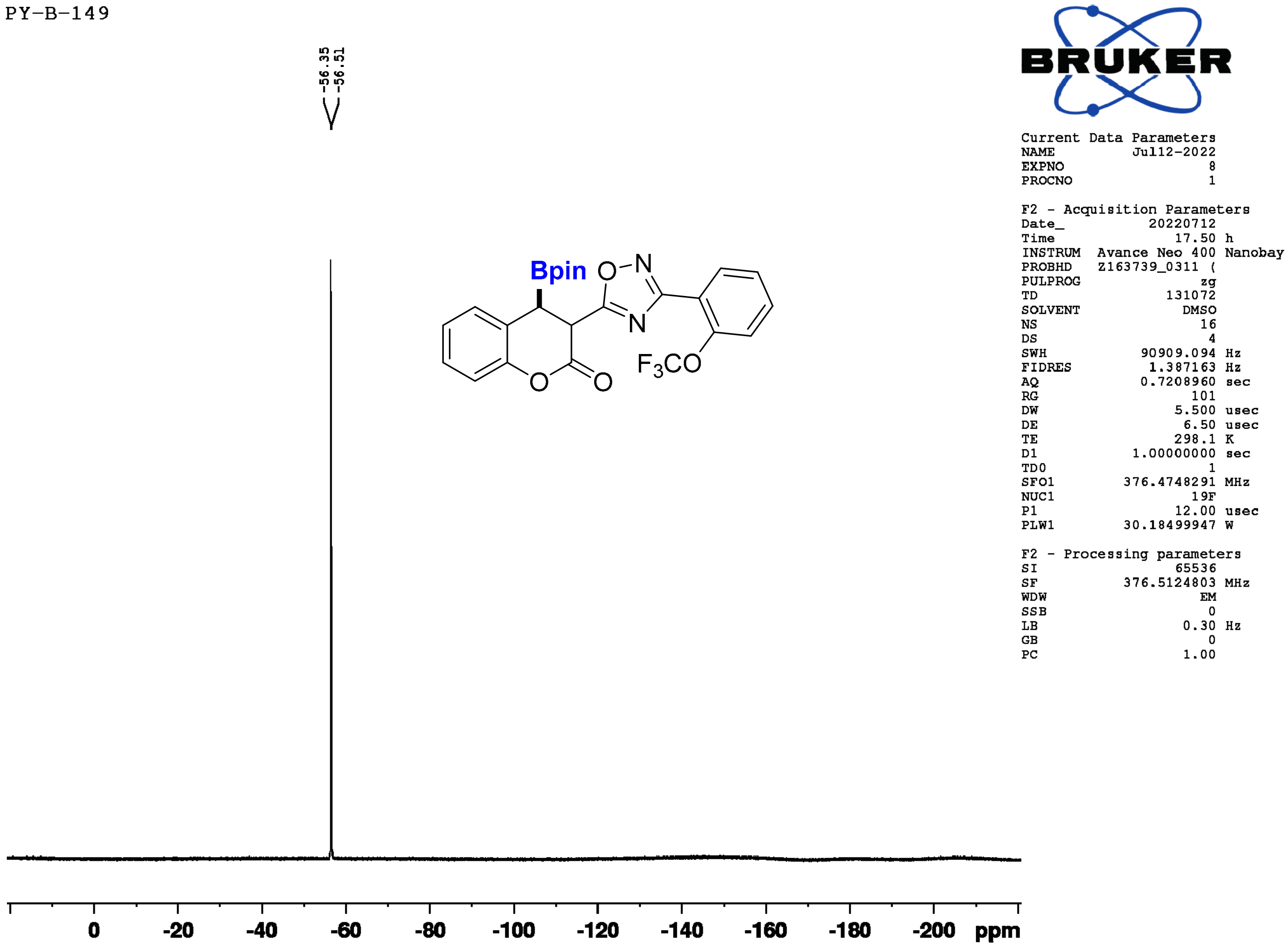

^{19}F NMR spectrum of **7b**

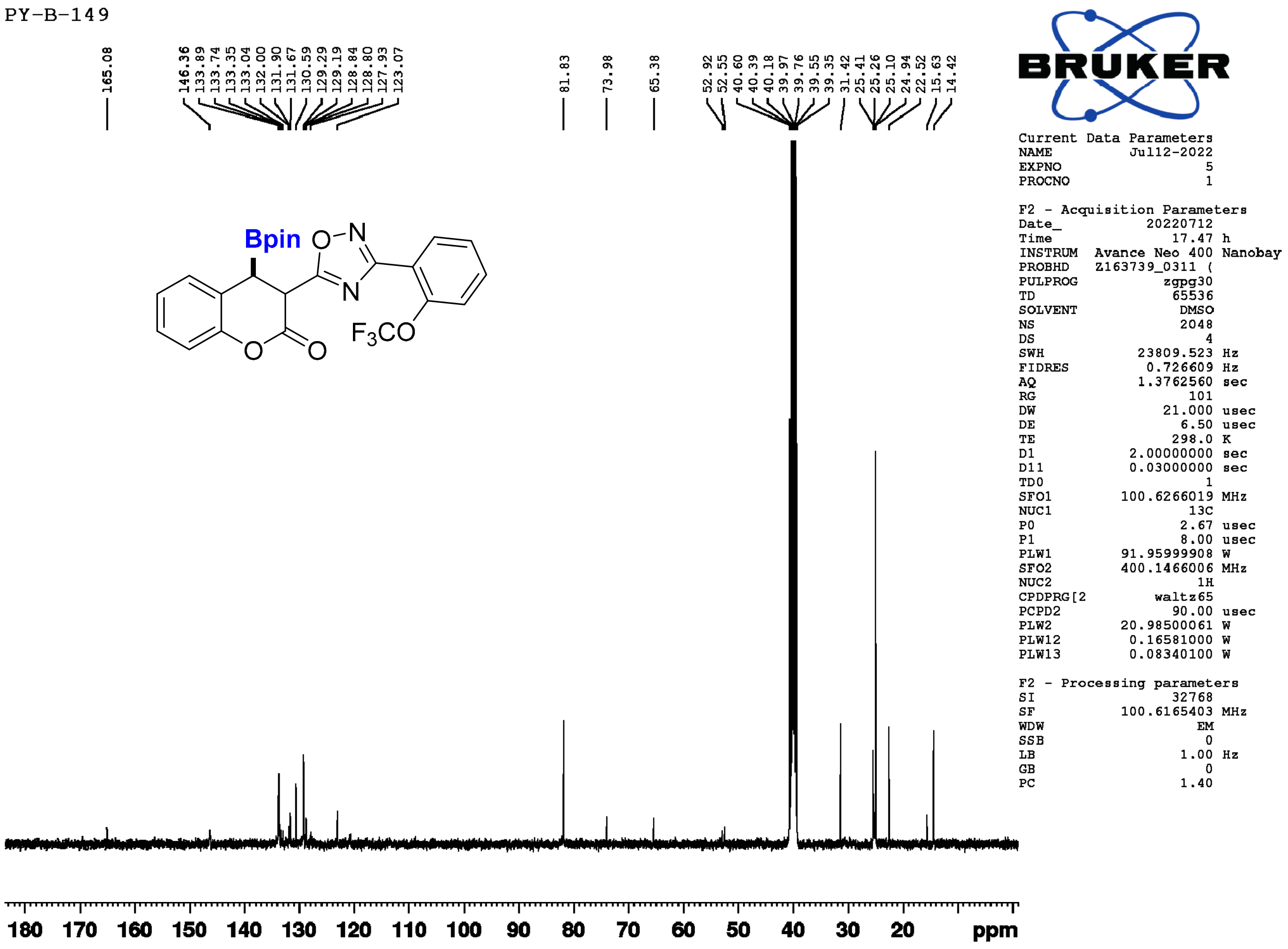

^{13}C NMR spectrum of **7b**

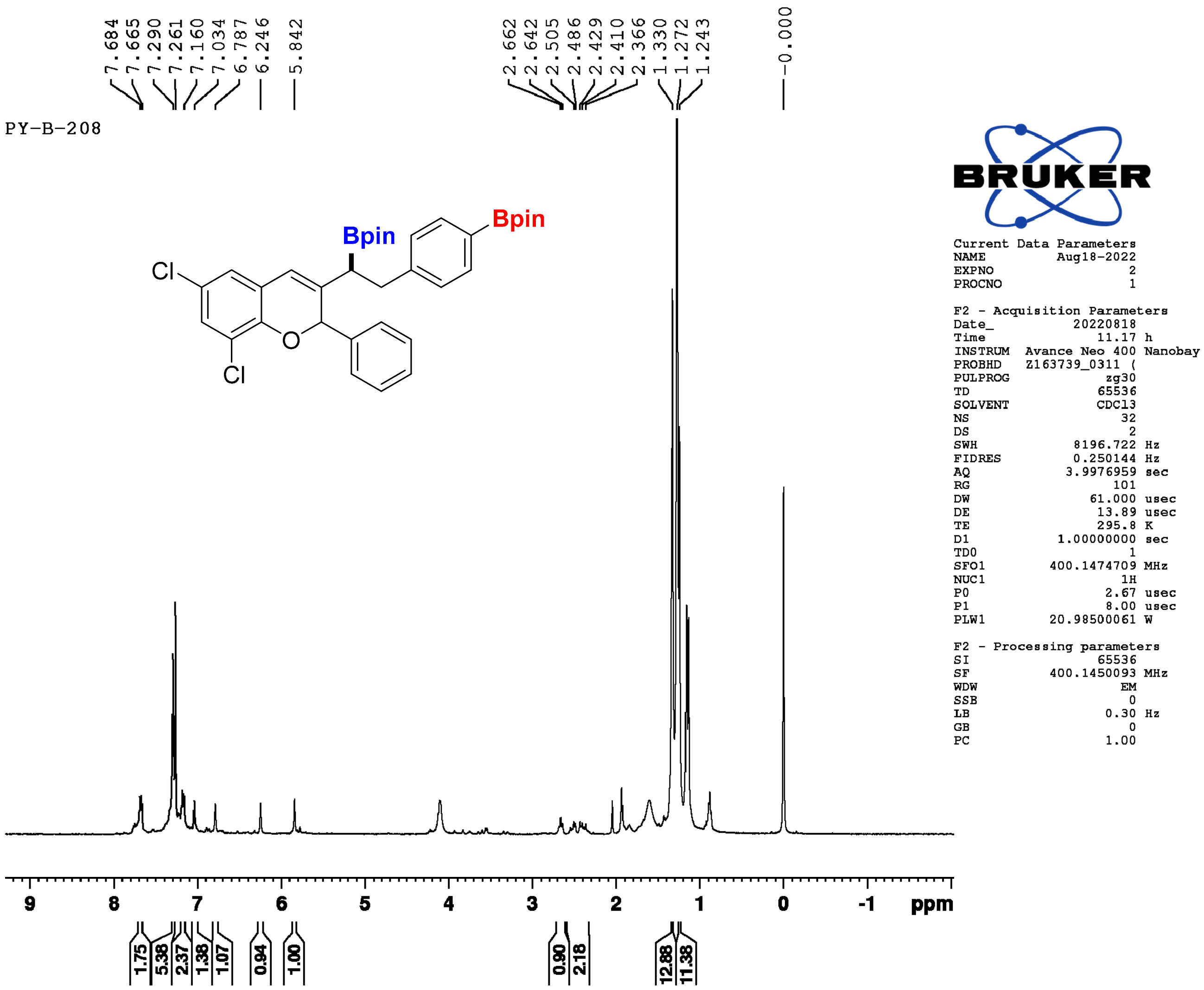

^{1}H NMR spectrum of **7c**

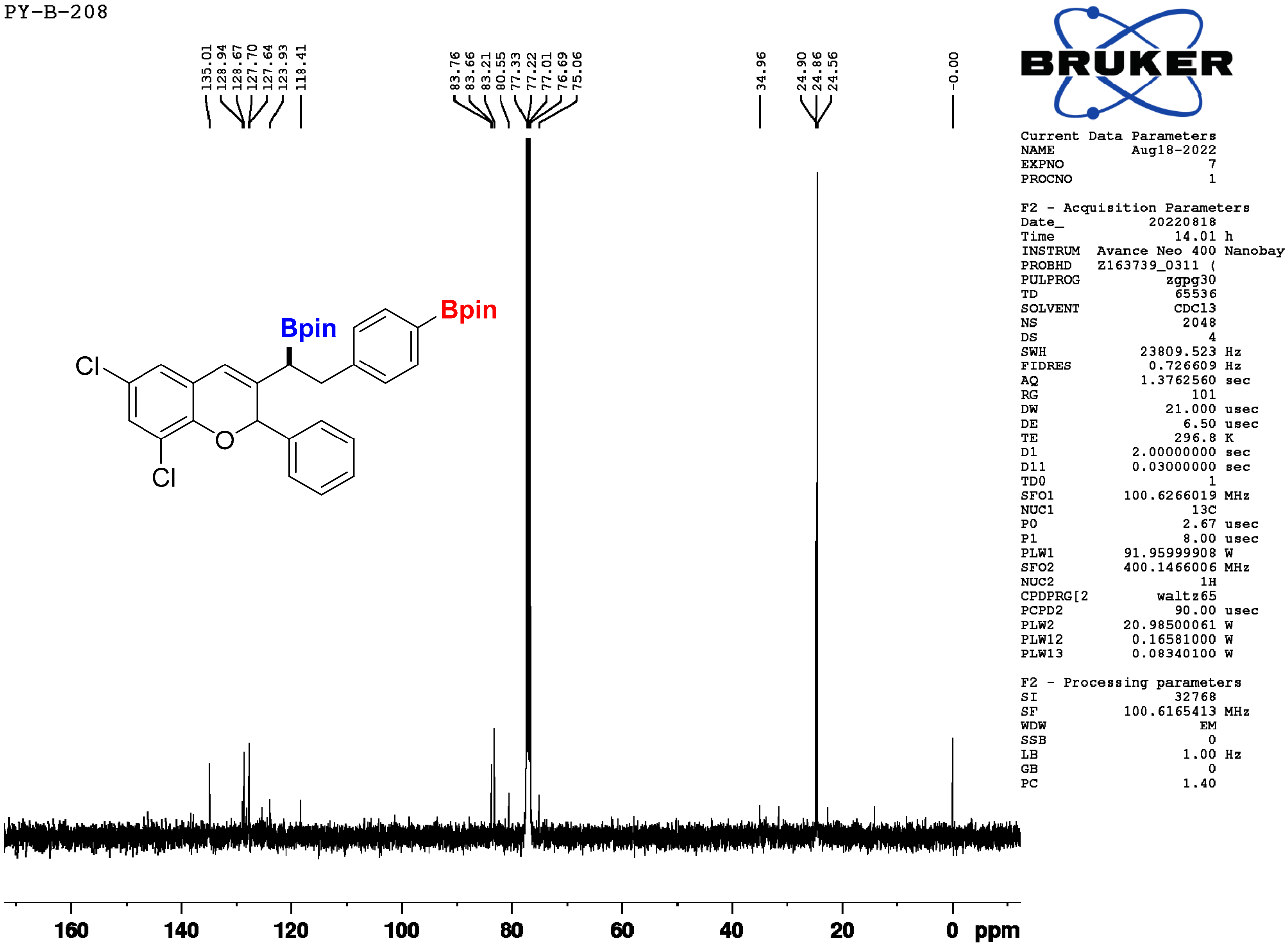

^{13}C NMR spectrum of **7c**

Spectrum View - PY-B-222.d

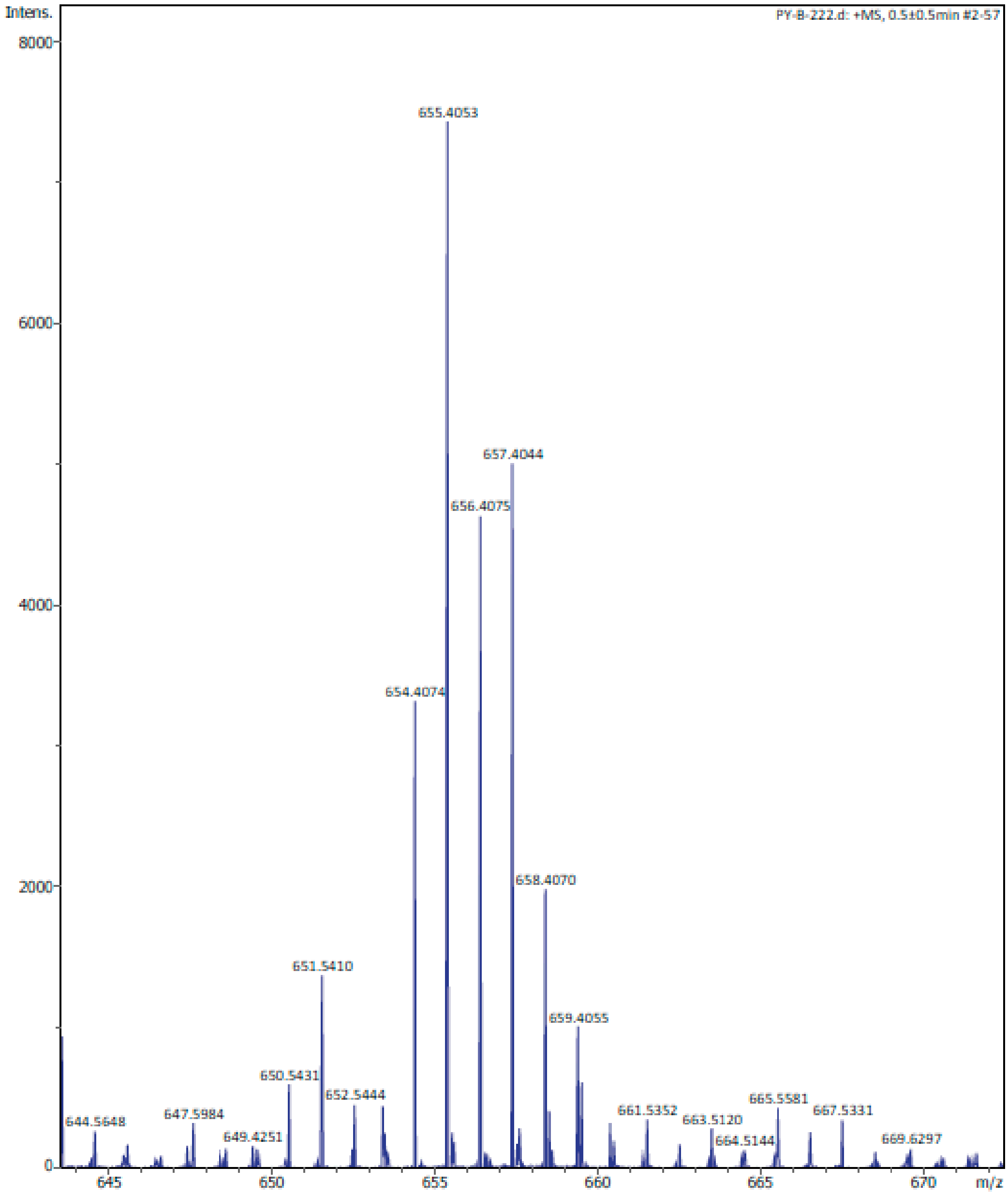

Mass spectrum of **7c**
